# Effects of machine perfusion strategies on different donor types in liver transplantation: a systematic review and meta-analysis

**DOI:** 10.1097/JS9.0000000000000661

**Published:** 2023-08-11

**Authors:** Aijun Liang, Weiye Cheng, Peihua Cao, Shaoru Cai, Linya Zhang, Kebo Zhong, Yu Nie

**Affiliations:** a General Surgery Center, Department of Hepatobiliary Surgery II, Guangdong Provincial Research Center for Artificial Organ and Tissue Engineering, Guangzhou Clinical Research and Transformation Center for Artificial Liver, Institute of Regenerative Medicine, Zhujiang Hospital, Southern Medical University,Guangzhou, Guangdong Province, China.; b Clinical Research Center, Zhujiang Hospital; c Department of Biostatistics, School of Public Health, Southern Medical University, Guangzhou, Guangdong, People’s Republic of China

**Keywords:** extended criteria donors, liver transplantation, machine perfusion, marginal grafts

## Abstract

**Background:**

The increasing use of extended criteria donors (ECD) sets higher requirements for graft preservation. Machine perfusion (MP) improves orthotopic liver transplantation (OLT) outcomes, but its effects on different donor types remains unclear. The authors’ aim was to assess the effects of hypothermic machine perfusion (HMP), normothermic machine perfusion (NMP), or normothermic regional perfusion (NRP) versus static cold storage (SCS) on different donor types.

**Materials and methods:**

A literature search comparing the efficacy of MP versus SCS in PubMed, Cochrane, and EMBASE database was conducted. A meta-analysis was performed to obtain pooled effects of MP on ECD, donation after circulatory death (DCD), and donor after brainstem death.

**Results:**

Thirty nine studies were included (nine randomized controlled trials and 30 cohort studies). Compared with SCS, HMP significantly reduced the risk of non-anastomotic biliary stricture (NAS) [odds ratio (OR) 0.43, 95% confidence interval (CI) 0.26–0.72], major complications (OR 0.55, 95% CI 0.39–0.78), and early allograft dysfunction (EAD) (OR 0.46, 95% CI 0.32–0.65) and improved 1-year graft survival (OR 2.36, 95% CI 1.55–3.62) in ECD-OLT. HMP also reduced primary non-function (PNF) (OR 0.40, 95% CI 0.18–0.92) and acute rejection (OR 0.62, 95% CI 0.40–0.97). NMP only reduced major complications in ECD-OLT (OR 0.56, 95% CI 0.34–0.94), without favorable effects on other complications and survival. NRP lowered the overall risk of NAS (OR 0.27, 95% CI 0.11–0.68), PNF (OR 0.43, 95% CI 0.22–0.85), and EAD (OR 0.58, 95% CI 0.42–0.80) and meanwhile improved 1-year graft survival (OR 2.40, 95% CI 1.65–3.49) in control DCD-OLT.

**Conclusions:**

HMP might currently be considered for marginal livers as it comprehensively improves ECD-OLT outcomes. NMP assists some outcomes in ECD-OLT, but more evidence regarding NMP-ECD is warranted. NRP significantly improves DCD-OLT outcomes and is recommended where longer non-touch periods exist.

## Introduction

Highlights

- We accurately evaluate the benefits of different machine perfusion (MP) methods [including hypothermic machine perfusion (HMP), normothermic machine perfusion (NMP), and normothermic regional perfusion (NRP)] on different donor types.
- HMP significantly reduces the risk of non-anastomotic biliary stricture (NAS), major complications, and early allograft dysfunction (EAD), meanwhile improves 1-year graft survival in all DBD-OLT (donor after brainstem death-orthotopic liver transplantation), DCD-OLT (donation after circulatory death-OLT), and ECD-OLT (extended criteria donor-OLT).
- NMP only reduces the risk of major complications in ECD-OLT, and failed to improve survival in all donor types.
- NRP lowers the occurrences of NAS, PNF (primary non-function), and EAD, meanwhile markedly elevates 1-year graft and patient survival rates in cDCD-OLT (controlled DCD-OLT).

Orthotopic liver transplantation (OLT) is the only well-established treatment for end-stage liver disease, but many patients died on the waiting list due to severe shortage of donors. To address shortfalls in the supply of standard criteria donors, many transplant centers have progressively extended the criteria to accept marginal livers or extended criteria donors (ECD)^1,2^, which could additionally increase the donor pool by up to 20–50% in some centers. However, ECD livers are frequently associated with high risk of postoperative short-term and long-term complications, as high-risk donors are more vulnerable to ischemia-reperfusion injury (IRI)^3^. Especially, primary non-function (PNF), early graft failure, and non-anastomotic biliary stricture (NAS), that are the frequent cause of early patient death, have always been the Achilles heel for ECD utilization. The discrepancy between the increasing need for ECD and the high perioperative complications of ECD has become a hard nut to crack in OLT. Therefore, new strategies for safe use of ECD grafts are urgently needed.

Static cold storage (SCS) remains the gold standard for organ preservation worldwide due to its simplicity, low cost, and convenience for transport. However, it cannot satisfy the requirements of high-quality preservation for ECD grafts. To expand the donor pool and mitigate the drawbacks of ECD, machine perfusion (MP) has emerged as a promising preservation method since it can greatly alleviate IRI and thus reduce the discarded rates^4^. By allowing for ex-situ nutrients supply, organ resuscitation, and viability evaluation, all hypothermic machine perfusion (HMP), normothermic machine perfusion (NMP), and normothermic regional perfusion (NRP) have been shown to outperform SCS through modulating IRI^2,5,6^, repairing liver grafts, and thus reducing post-OLT complications, so more margin donors can be saved to combat the organ shortages.

In some countries, donation after circulatory death (DCD) donors has extremely high discarding rates due to long functional warm ischemia time (WIT) under ethical principle and requirements. To address this, the VITTAL trial has attempted to use NMP to rescue declined livers to push the boundaries in utilization of high-risk grafts, but the outcomes in DCD grafts were not satisfactory^7^. The concept of NRP was introduced to reduce IRI during non-touching period before procurement. Recent studies suggested NRP as a safe alternative to in-situ cooling and rapid procurement^8^, which could also reduce certain post-transplant complications such as NAS and early allograft dysfunction (EAD)^8–10^. However, these results are not so convincing as they are inconsistent.

All HMP, NMP, and NRP could theoretically improve OLT outcomes through protecting grafts from IRI. However, more convincing evidence is still needed. Currently, the number of randomized controlled trials (RCTs) on MP is still limited, and heterogeneity makes the results remain controversial. Previous meta-analyses have demonstrated the importance of MP in reducing EAD and NAS^4,10^. However, these meta-analyses are vague about the effectiveness of MP on different graft types. The preferred MP method for certain type of donor remains unclear, especially for ECD. These add to confusion in donor grafts management and cause unnecessary medical waste. In recent 2 years, a considerable number of high-quality researches on MP are updated. It is needed to summarize and confirm the selection criteria of different MP strategies for different donor types, especially for ECD.

The aim of this study was to appraise and condense the available literature reporting OLT outcomes when comparing HMP, NMP, and NRP with SCS to (i) assess the effects of different MP strategies on improving OLT outcomes in different donor types and (ii) preliminarily explore the preferred MP strategy for ECD grafts.

## Methods

This systematic review and meta-analysis was conducted in April 2023 on the basis of the PRISMA^11^ (Preferred Reporting Items for Systematic Reviews and Meta-Analyses recommendations) (Supplemental Digital Content 1, http://links.lww.com/JS9/A883) and AMSTAR (Assessing the methodological quality of systematic reviews) Guidelines^12^ (Supplemental Digital Content 2, http://links.lww.com/JS9/A884).

### Search strategy

The literature search was carried out on PubMed, Cochrane Library, EMBASE, ClinicalTrials.gov, and Web of Science, using the following search algorithm ‘(machine preservation OR machine perfusion OR normothermic machine perfusion OR NMP OR hypothermic machine perfusion OR HMP OR hypothermic oxygenated machine perfusion OR HOPE OR ex vivo machine perfusion OR normothermic regional perfusion OR NRP) AND MeSH terms (liver)’ in ‘All fields’. No language or date restrictions were applied. The reference lists of selected studies and previous reviews were manually reviewed to identify all relevant articles. Abstracts were screened and full-text articles were evaluated for eligibility by two independent reviewers. If a disagreement occurred between the reviewers, the senior author was consulted to reach a final decision together.

### Inclusion and exclusion criteria

Adult OLT studies (including RCT, case–control studies, prospective and retrospective cohort studies) comparing the outcomes of HMP, NMP, or NRP versus SCS were included.

Studies were excluded if: (a) articles with less than 10 transplanted livers; (b) studies using more than one perfusion technique [e.g. sequential hypothermic oxygenated perfusion (HOPE) and NMP]; (c) studies contained living or split donor or multivisceral transplantation or simultaneous liver–kidney transplantation or pediatric transplantation; (d) studies without controls or the control group was not SCS; (e) overlapping studies from the same center; (f) articles without the interest outcomes; (g) animal experiments, reviews, editorials, letters or case reports, book chapters, and abstracts.

### Outcomes and definitions

The primary outcomes extracted were NAS, major complications, EAD, and 1-year patient and graft survival. Secondary outcomes were acute cellular rejection (ACR), PNF, post-reperfusion syndrome (PRS), acute kidney injury (AKI), renal replacement therapy (RRT), and hepatic artery thrombosis (HAT).

NAS was defined as any irregularity or narrowing of the intrahepatic or extrahepatic donor bile ducts within 12 months following OLT, and it must be documented by Endoscopic Retrograde Cholangiopancreatography, percutaneous transhepatic cholangiogram, surgically placed biliary catheter, or magnetic resonance cholangiopancreatogram^13^. Major complication defined as major complications defined by a Clavien–Dindo grade ≥3. ECD were defined as donors with hemodynamic deterioration, donor age >65 years, donor body mass index >30 kg/m^2^, serum bilirubin >3 mg/dl, Aspartate Aminotransferase or Alanine Aminotransferase above three times the upper reference threshold, sodium >165 mmol/l, intensive care unit (ICU) stay >7 days, steatosis >40%, cold ischemic time (CIT) >12 h^1^. Detailed definitions of primary and secondary endpoints can be found in the Supplementary Materials (Supplemental Digital Content 3, http://links.lww.com/JS9/A885).

### Data extraction

Data extraction was performed using a predetermined Microsoft Excel template. The data included characteristics of the study (the first author, publication year, study design, study period, sample size), donor and recipient characteristics (type of grafts, donor and recipient age, lab model for end-stage liver disease score), MP parameters [perfusion types, function warm ischemic time (fWIT), CIT, MP time, total preservation time cannulation route, perfusion solution, device, temperature, active oxygenation, pressure], and the outcomes above. Any differences in data extraction were settled by discussion and consultation with the senior authors.

### Risk of bias

Two reviewers determined independently the risk of bias according to the Risk of Bias in Nonrandomized Studies of Interventions tool for cohort studies and the Risk of Bias tool 2 (RoB2) or RCTs.

### Data analysis

Results were expressed as odds ratio (OR) with 95% confidence intervals (CI). Heterogeneity was analyzed using Cochran’s *Q* and the *I* ^2^ statistics. A meta-analysis was conducted using random-effects model or fixed-effects model, with fixed-effects model used if heterogeneity was less than 50%. First, we focused on the effects of HMP, NMP, or NRP on all donor types. Subsequently, subgroup analysis was used to explore the effects on ECD, DCD, or donor after brainstem death (DBD). Possible sources of heterogeneity were explored by subgroup analysis and sensitivity analysis. When analyzing NRP studies, we further divided DCD into controlled DCD (cDCD) and uncontrolled DCD (uDCD). Finally, we conducted the meta-analysis using only RCTs to verify the stability of the results. The statistical package R version 4.2.0 was used for statistical analysis. A two-tailed *P*<0.05 was considered as statistically significant.

## Results

**Figure 1:**
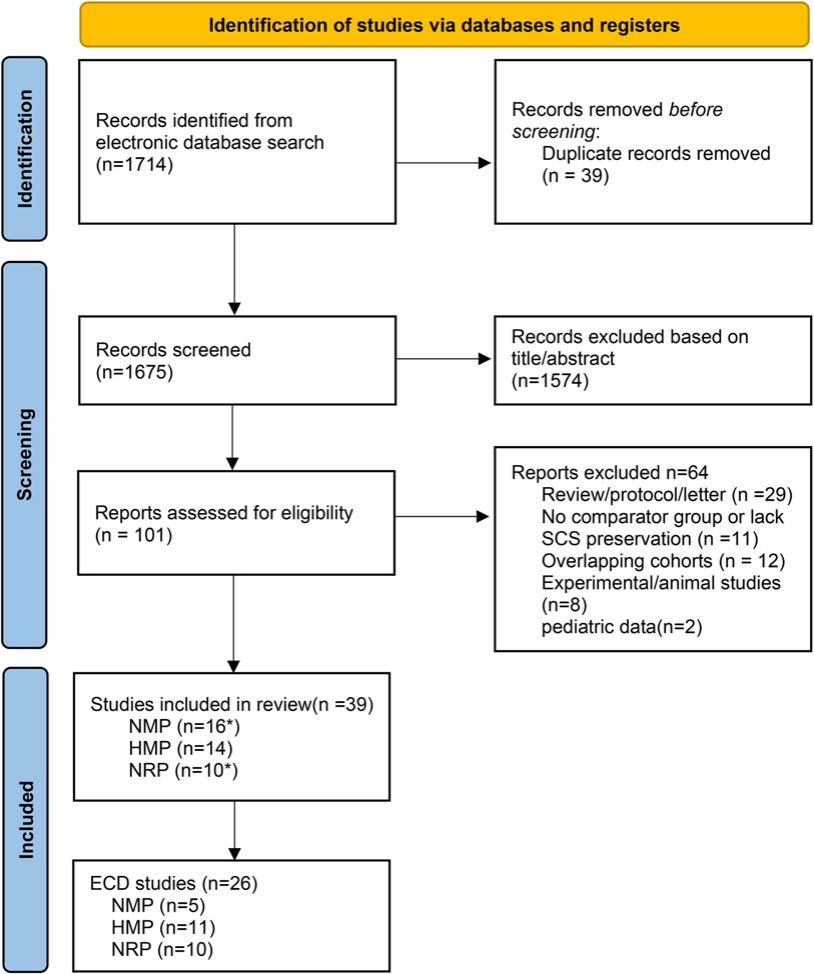
PRISMA (Preferred Reporting Items for Systemic Reviews and Meta-Analyses) flow diagram.

Literature search finally identified 1714 records, and 101 full-text articles were assessed for eligibility. In total, 39 studies^1,2,5–8,14–46^ were eligible in the systematic review, including nine RCTs^1,2,6,14,22,23,33,44,46^ and 30 cohort studies^5,7,8,15–21,24–32,34–42,44,45,47^. A total of 6254 livers were included: 3970 SCS (control group), 608 grafts with HMP, 709 with NMP, and 968 with NRP, of which 1122 livers were from RCTs. Study information, recipient and donor demographics, and perfusion characteristics are summarized in Tables S3–S7 (Supplemental Digital Content 3, http://links.lww.com/JS9/A885). Detailed distribution of baseline characteristics is described in Supplementary Page 1 (Supplemental Digital Content 3, http://links.lww.com/JS9/A885). A PRISMA flow chart for included studies is presented in Figure 1. The risk of bias evaluation is reported in Tables S1 (Supplemental Digital Content 3, http://links.lww.com/JS9/A885) and S2 (Supplemental Digital Content 3, http://links.lww.com/JS9/A885). Overall, all RCTs had a low risk of bias. Non-randomized cohort studies had at least moderate risk bias [Tables S1 and S2 (Supplemental Digital Content 3, http://links.lww.com/JS9/A885)].

## Hypothermic machine perfusion

### HMP’s performance on all-donor-typed OLT outcomes

Fourteen HMP studies^1,2,18,24–30,32,33,35,46^ were involved in meta-analyses, including four RCTs^1,2,33,46^. No significant heterogeneity was observed. For overall effects on all donor types, HMP was associated with significant improvements in NAS (OR 0.43, 95% CI 0.26–0.70, *P*<0.01), major complication (OR 0.62, 95% CI 0.46–0.84, *P*<0.01), and EAD (OR 0.39, 95% CI 0.30–0.53, *P*<0.01). One-year graft survival (OR 2.31, 95% CI 1.54–3.45, *P*<0.01) and 1-year patient survival (OR 1.79, 95% CI 1.15–2.79, *P*=0.01) were significantly improved in HMP compared with SCS [Figs S1 (Supplemental Digital Content 3, http://links.lww.com/JS9/A885) and S2 (Supplemental Digital Content 3, http://links.lww.com/JS9/A885)].

### HMP’s performance on ECD-OLT outcomes

**Figure 2:**
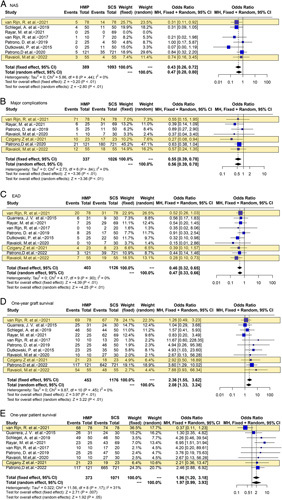
Forest plots on NAS (A), major complications (B), EAD (C), 1-year graft survival (D), and 1-year patient survival (E) in ECD-OLT after HMP compared with SCS. CI, confidence interval; ECD, extended/expanded criteria donor; EAD, early allograft dysfunction; HMP, hypothermic machine perfusion; NAS, non-anastomotic biliary stricture; SCS, static cold storage. RCT data are highlighted in yellow.

Eleven studies^1,2,18,25–30,35,46^ reported the effects of HMP on ECD, including three RCTs^1,2,46^. HMP remarkably reduced the risk of NAS in ECD-OLT compared with SCS (OR 0.43, 95% CI 0.26–0.72, *P*<0.01); this benefit was maintained significant in major complications (OR 0.55, 95% CI 0.39–0.78, *P*<0.01) and EAD (OR 0.46, 95% CI 0.32–0.65, *P*<0.01). HMP was also significantly associated with improved 1-year graft survival (OR 2.36, 95% CI 1.55–3.62, *P*<0.01) and 1-year patient survival (OR 1.96, 95% CI 1.20–3.18, *P*=0.007) compared with SCS in ECD subgroup (Fig. 2).

### HMP’s performance on DCD-OLT outcomes

**Figure 3:**
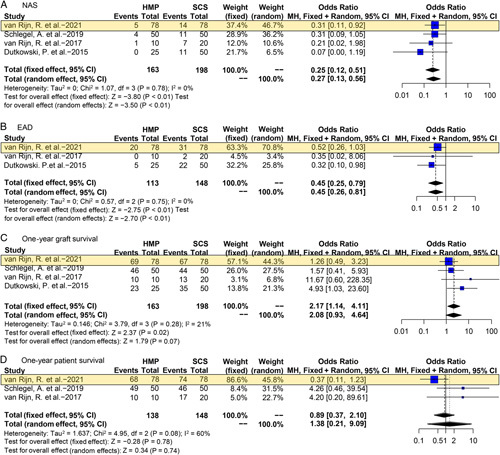
Forest plots on NAS (A), EAD (B), 1-year graft survival (C), and 1-year patient survival (D) in DCD-OLT after HMP compared with SCS. CI, confidence interval; DCD, donation after circulatory death; EAD, early allograft dysfunction; HMP, hypothermic machine perfusion; NAS, non-anastomotic biliary stricture; SCS, static cold storage. RCT data are highlighted in yellow.

Four studies reported HMP-DCD outcomes^2,26,28,30^ and one was RCT^2^. Compared with SCS-DCD, risk of NAS was significantly decreased in HMP-DCD group (OR 0.25 95% CI 0.12–0.51, *P*<0.01); and this benefit was maintained in EAD (OR 0.45, 95% CI 0.25–0.79, *P*<0.01) (Fig. 3). Since lack of enough data for major complications, analysis for this endpoint cannot be conducted. In van Rijn’s RCT^2^, there were no significant differences in incidence of various grades of major complications between HMP-DCD and SCS-DCD (*P*>0.05). One-year graft survival was significantly improved in HMP-DCD (OR 2.17, 95% CI 1.14–4.11, *P*=0.02). However, there was no significant difference in 1-year patient survival between HMP-DCD and SCS-DCD (OR 1.38, 95% CI 0.21–9.09, *P*=0.74) (Fig. 3).

### HMP’s performance on DBD-OLT outcomes

Nine articles^1,24,27,29,31–33,35,46^ included HMP-DBD and three were RCTs^3,33,46^. For DBD grafts, no difference was found on NAS between HMP and SCS (OR 0.76, 95% CI 0.38–1.54, *P*=0.45). Compared with SCS, HMP remarkably reduced the risk of postoperative major complications (OR 0.63 95% CI 0.46–0.85, *P*<0.01) and EAD (OR 0.37, 95% CI 0.26–0.52, *P*<0.01). HMP was significantly associated with improved 1-year graft survival (OR 2.76, 95% CI 1.54–4.93, *P*<0.01) and 1-year patient survival (OR 2.49, 95% CI 1.38–4.52, *P*<0.01) in DBD grafts [Fig. S3 (Supplemental Digital Content 3, http://links.lww.com/JS9/A885)].

## Normothermic machine perfusion

### NMP’s performance on all-donor-typed OLT outcomes

A total of 16^5–7,14–23,43–45^ studies reported the effects of NMP on OLT outcomes, including five RCTs^6,14,22,23,43^. Overall risk of NAS (OR 0.55, 95% CI 0.36–0.84, *P*<0.01) and EAD (OR 0.55, 95% CI 0.34–0.88, *P*=0.01) was significantly lower in NMP than SCS, but no difference was observed in major complications (OR 0.65, 95% CI 0.32–1.31, *P*=0.23). High heterogeneity was observed among the included studies for these endpoints [Fig. S4 (Supplemental Digital Content 3, http://links.lww.com/JS9/A885)]. No differences were found in 1-year graft survival (OR 1.11, 95% CI 0.75–1.65, *P*=0.60) and patient survival (OR 1.13, 95% CI 0.74–1.71, *P*=0.57) [Figs S4 (Supplemental Digital Content 3, http://links.lww.com/JS9/A885) and S5 (Supplemental Digital Content 3, http://links.lww.com/JS9/A885)].

### NMP’s performance on ECD-OLT outcomes

**Figure 4:**
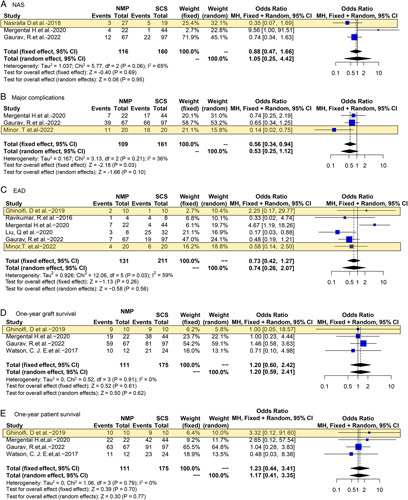
Forest plots on NAS (A), major complications (B), EAD (C), 1-year graft survival (D), and 1-year patient survival (E) in ECD-OLT after NMP compared with SCS. CI, confidence interval; ECD, extended/expanded criteria donor; EAD, early allograft dysfunction; NAS, non-anastomotic biliary stricture; NMP, normothermic machine perfusion; SCS, static cold storage. RCT data are highlighted in yellow.

Eight^6,7,14,18–21,23^ NMP studies reported the outcomes of ECD-OLT. The risk of NAS (OR 1.05, 95% CI 0.25–4.42, *P*=0.95) and EAD (OR 0.74, 95% CI 0.26–2.07, *P*=0.56) was similar between NMP-ECD and SCS-ECD. Instead, major complications were significantly reduced in NMP-ECD (OR 0.56, 95% CI 0.34–0.94, *P*=0.03). No differences were observed in 1-year graft survival (OR 1.20, 95% CI 0.60–2.42, *P*=0.61) and 1-year patient survival (OR 1.23, 95% CI 0.44–3.41, *P*=0.70) between NMP and SCS (Fig. 4).

### NMP’s performance on DCD-OLT outcomes

**Figure 5:**
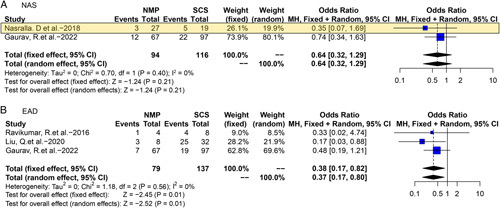
Forest plots on NAS (A) and EAD (B) in DCD-OLT after NMP compared with SCS. CI, confidence interval; DCD, donation after circulatory death; EAD, early allograft dysfunction; NMP, normothermic machine perfusion; NAS, non-anastomotic biliary stricture; SCS, static cold storage. RCT data are highlighted in yellow.

Four studies reported the outcomes of NMP-DCD^6,18–20^. Only NAS and EAD were included in meta-analysis due to data accessibility. Pooled analysis of two studies^6,20^ showed that NAS risk was similar between NMP-DCD and SCS-DCD (OR 0.64, 95% CI 0.32–1.29, *P*=0.21). In the meta-analysis of three studies^18–20^, EAD was significantly reduced in NMP-DCD (OR 0.38, 95% CI 0.17–0.82, *P*=0.01) (Fig. 5).

### NMP’s performance on DBD-OLT outcomes

Five studies^5,14,15,43,45^ reported NMP-DBD outcomes and two were RCTs^14,43^. Only EAD, 1-year graft survival, and 1-year patient survival had enough data for meta-analysis. Compared with SCS-DBD, there was no significant difference in EAD (OR 0.41, 95% CI 0.13–1.32, *P*=0.13) in NMP-DBD. No statistical difference was found in 1-year graft survival (OR 1.38, 95% CI 0.60–3.18, *P*=0.46) and 1-year patient survival between NMP-DBD and SCS-DBD (OR 1.55, 95% CI 0.66–3.63, *P*=0.31) [Fig. S6 (Supplemental Digital Content 3, http://links.lww.com/JS9/A885)].

### NMP’s performance on studies including both DBD and DCD

Eight NMP studies^6,7,16,18,19,21,22,44^ included both DBD and DCD grafts. As these studies did not report the outcomes separately according donor types, we specifically performed a subgroup analysis of NMP studies including both DBD and DCD. There were no differences in NAS (OR 0.65, 95% CI 0.18–2.37, *P*=0.52), major complications (OR 0.63, 95% CI 0.23–1.68, *P*=0.35), EAD (OR 0.70, 95% CI 0.37–1.32, *P*=0.27), 1-year graft survival (OR 0.96, 95% CI 0.57–1.63, *P*=0.88), and 1-year patient survival (OR 1.01, 95% CI 0.60–1.69, *P*=0.97) between NMP and SCS in this subgroup [Fig. S7 (Supplemental Digital Content 3, http://links.lww.com/JS9/A885)].

## Normothermic regional perfusion

### NRP’s performance on all-donor-typed OLT outcomes

Though ten^8,20,34,36–42^ studies investigated the impacts of NRP on OLT outcomes, currently no RCTs were available on this topic. Seven studies^8,20,34,36,38,41,42^ investigated NRP-cDCD grafts, with three^8,20,41^ using SCS-cDCD as controls and the remaining four^34,36,37,42^ using SCS-DBD as controls. NRP-uDCD was investigated in three studies^37,39,40^ with SCS-DBD as control.

In pooled analysis [Figs S8 (Supplemental Digital Content 3, http://links.lww.com/JS9/A885) and S9 (Supplemental Digital Content 3, http://links.lww.com/JS9/A885)], NRP significantly reduced the overall risk of EAD (OR 0.69, 95% CI 0.54–0.90, *P*<0.01). However, there were no significant differences in NAS (OR 0.97, 95% CI 0.17–5.46, *P*=0.98) and major complications (OR 0.56, 95% CI 0.06–4.93, *P*=0.60) between NRP and SCS. One-year graft survival (OR 1.44, 95% CI 0.64–3.25, *P*=0.38) and 1-year patient survival (OR 1.15, 95% CI 0.53–2.46, *P*=0.73) were also similar between NRR and SCS. The heterogeneity was high in these outcomes [Figs S8 (Supplemental Digital Content 3, http://links.lww.com/JS9/A885) and S9 (Supplemental Digital Content 3, http://links.lww.com/JS9/A885)].

### NRP’s performance on cDCD outcomes

As both SCS-cDCD and SCS-DBD were used as controls, subgroup analysis was performed in NRP-cDCD studies.

**Figure 6:**
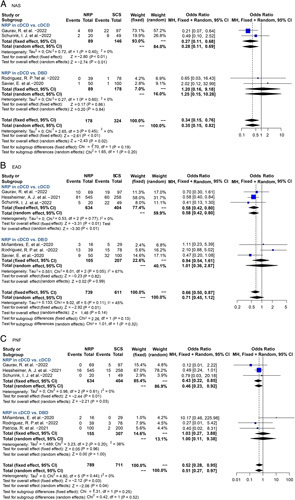
Forest plots on NAS (A), EAD (B), and PNF (C) in controlled DCD-OLT after NRP compared with SCS. cDCD, controlled donation after circulatory death; CI, confidence interval; EAD, early allograft dysfunction; NAS, non-anastomotic biliary stricture; NMP, normothermic machine perfusion; PNF, primary non-function; SCS, static cold storage.

**Figure 7:**
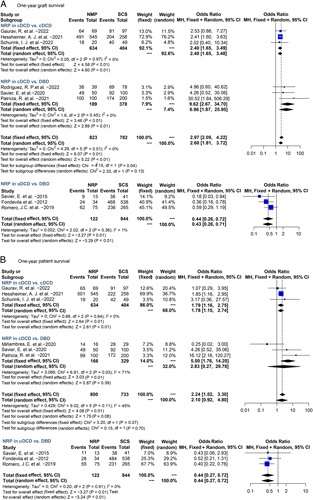
Forest plots on 1-year graft survival (A) and 1-year patient survival (B) in controlled DCD and uncontrolled DCD-OLT after NRP compared with SCS. cDCD, controlled donation after circulatory death; CI, confidence interval; SCS, static cold storage; NRP, normothermic regional perfusion; uDCD, uncontrolled donation after circulatory death.

Compared with SCS-cDCD, NRP-cDCD had significant lower risk of NAS (OR 0.27, 95% CI 0.11–0.68, *P*<0.01), EAD (OR 0.58, 95% CI 0.42–0.80, *P*<0.01), and PNF (OR 0.43, 95% CI 0.22–0.85, *P*=0.01) (Fig. 6). NRP-cDCD also had significantly improved 1-year graft survival (OR 2.40, 95% CI 1.65–3.49, *P*<0.01) and patient survival (OR 1.79, 95% CI 1.16–2.75, *P*<0.01) (Figs 6, 7).

Compared with SCS-DBD, NAS (OR 1.20, 95% CI 0.16–9.18, *P*=0.86), EAD (OR 1.01, 95% CI 0.36–2.87, *P*=0.99), and PNF (OR 1.03, 95% CI 0.27–3.88, *P*=0.96) were similar in NRP-cDCD. One-year graft survival (OR 9.62, 95% CI 2.67–34.70, *P*<0.01) was significantly improved in NRP-cDCD, with no difference in 1-year patient survival (OR 2.83, 95% CI 0.27–29.78, *P*=0.39) (Figs 6, 7).

### NRP’s performance on uDCD outcomes

There was insufficient data for meta-analysis of NAS, EAD, and PNF in NRP-uDCD grafts. Compared with SCS-uDCD, NRP-uDCD failed to achieve comparative 1-year graft survival (OR 0.44, 95% CI 0.26–0.72, *P*<0.01) and 1-year patient survival (OR 0.44, 95% CI 0.27–0.72, *P*<0.01) (Fig. 7).

### Results from RCTs

A subgroup analysis was performed using data from RCTs. Nine RCTs ^1,2,6,14,22,23,33^ were eligible in meta-analysis, including five NMP studies ^6,14,22,23,43^ and four HMP studies ^1,2,33,46^.

### RCT data for HMP

In subgroup analysis of HMP-RCTs, HMP was associated with a significant reduction in NAS (OR 0.40, 95% CI 0.18–0.89, *P*=0.03), EAD (OR 0.34, 95% CI 0.22–0.52, *P*<0.01), and major complications (OR 0.66, 95% CI 0.43–1, *P*=0.05) compared with SCS. HMP significantly improved 1-year graft survival (OR 2.01, 95% CI 1.06–3.81, *P*=0.03) but had no benefits on 1-year patient survival (OR 0.73, 95% CI 0.34–1.60, *P*=0.43) (Fig. S10 (Supplemental Digital Content 3, http://links.lww.com/JS9/A885)].

### RCT data for NMP

In subgroup analysis of NMP-RCTs, NMP significantly reduced NAS (OR 0.32, 95% CI 0.15–0.65, *P*<0.01), major complications (OR 0.38, 95% CI 0.23–0.63, *P*<0.01), and EAD (OR 0.41, 95% CI 0.27–0.60, *P*<0.01). However, NMP showed no benefits on 1-year graft survival (OR 0.86, 95% CI 0.31–2.37, *P*=0.76) and 1-year patient survival (OR 1.01, 95% CI 0.50–2.07, *P*=0.97) [Fig. S11 (Supplemental Digital Content 3, http://links.lww.com/JS9/A885)].

### Secondary outcomes

In a pooled analysis, HMP significantly decreased the risk of PNF (OR 0.40, 95% CI 0.18–0.92, *P*=0.03) and ACR (OR 0.62, 95% CI 0.40–0.97, *P*=0.02) and tended to reduce PRS (OR 0.42, 95% CI 0.15–1.16, *P*=0.09) and HAT (OR 0.59, 95% CI 0.30–1.16, *P*=0.12) compared to SCS [Figs S12 (Supplemental Digital Content 3, http://links.lww.com/JS9/A885) and S13 (Supplemental Digital Content 3, http://links.lww.com/JS9/A885)]. In a subgroup analysis of ACR, HMP significantly reduced ACR only in ECD-DCD grafts (OR 0.37, 95% CI 0.19–0.71, *P*<0.01) but not in ECD-DBD grafts (OR 0.75, 95% CI 0.36–1.57, *P*=0.44). No differences were observed in AKI and RRT between HMP and SCS (*P*>0.05).

NMP tended to reduce the risk of PNF (OR 0.36, 95% CI 0.12–1.04, *P*=0.06) but had no effects on reducing PRS, ACR, RRT, and HAT (*P*>0.05) [Figs S14 (Supplemental Digital Content 3, http://links.lww.com/JS9/A885) and S15 (Supplemental Digital Content 3, http://links.lww.com/JS9/A885)].

NRP significantly reduced the rates of ACR (OR 0.66, 95% CI 0.44–0.99, *P*=0.05) and HAT (OR 0.54, 95% CI 0.33–0.90, *P*=0.02) but had no effects on reducing PRS and RRT (*P*>0.05) [Fig. S16 (Supplemental Digital Content 3, http://links.lww.com/JS9/A885)].

## Discussion

MP is considered as the biggest breakthrough in OLT in the last decade, allowing for increasing organ utilization, prolonged preservation, organ reconditioning, and viability evaluation. Many lines of evidence have indicated MP’s superiority to SCS, but the best perfusion strategy for certain donor type remains unclear, especially for ECD grafts. To preliminarily evaluate the relative strength of different perfusion strategies (HMP, NMP, or NRP) on different donor types, we performed a meta-analysis according to DBD, DCD, and ECD. Our results show first that HMP, NMP, and NRP are all effective to improve OLT outcomes, but their protective effects vary to a certain degree on different donor types. Compared with SCS, HMP could comprehensively improve OLT outcomes, including reducing the risk of NAS, EAD, major complications, PNF, PRS, and ACR and meanwhile improve 1-year graft/patient survival, with the strongest effects seen in ECD. Second, NMP could reduce major complications in ECD and EAD in DCD. Third, NRP significantly reduced NAS, EAD, PNF, and HAT and improved 1-year graft survival in cDCD.

The issue of improving preservation quality is at the core of transplantation, especially for high-risk grafts. ECD are increasingly used due to severe organ shortage, but many ECD livers are still declined due to high risk of PNF or severe complications. MP has the potential to address this dilemma, and various protocols are currently investigated in clinical trials. However, MP’s benefits on ECD lack quantitative investigation, and the priority of different MP methods remains unknown. Our study first demonstrated that there is an overall improvement in HMP-OLT outcomes, including reducing various short-term complications and improving graft survival. This effect is significant in all DBD, DCD, and ECD, with the strongest effects seen with ECD. PNF and NAS are two critical IRI-related complications, which could directly reflect the preservation quality. Our study first showed that HMP significantly reduced PNF risk by 60% [OR 0.40, *P* =0.03, Fig. S12 (Supplemental Digital Content 3, http://links.lww.com/JS9/A885)] and NAS risk by 57% [all donor types, OR 0.43, *P*<0.01, Fig. S1 (Supplemental Digital Content 3, http://links.lww.com/JS9/A885)] compared with SCS, indicating HMP’s excellent effects against IRI. More interestingly and beyond our expectation, we also found HMP’s significant effects on reducing ACR [OR 0.62, *P*=0.02, Fig. S12 (Supplemental Digital Content 3, http://links.lww.com/JS9/A885)], which was consistent with Maspero’s findings^48^. Better still, our data further suggested that HMP’s benefits on ACR were only limited in DCD (OR 0.37, *P*<0.01) but not in DBD and ECD-DBD grafts (*P*>0.05), which needs further study to clarify this phenomenon. Graft survival is another important endpoint for MP’s trials. In our study, HMP significantly improved 1-year graft survival in all DBD, DCD, and ECD subgroups, demonstrating its long-lasting and outstanding protection on long-term outcomes. In Patrono’s study^35^, HMP is even associated with improved 5-year overall graft survival in elderly donors (>75 years old). A noteworthy study by Muller *et al*.^49^ highlights the significance of flavin mononucleotide in HMP setting as a reliable marker to determine the ‘transplantability’ and facilitate proper clinical decisions regarding the acceptance of high-risk grafts. Taken these results together, HMP has the potential to be the preferred preservation strategy for DCD or ECD grafts.

NMP is a landmark development in overcoming logistical challenges in OLT. NMP can not only resuscitate grafts to reduce IRI but also allow to facilitate viability assessment and decision-making of marginal grafts. According to Nasralla’s^6^ RCT and VITTAL clinical trial, NMP has resulted in a 50% decrease in discard rate and a 20% increase in transplanted liver in comparison to SCS. And this benefit is achieved on accepting high-risk donors that have previously been declined by conventional concepts. However, high rates of NAS in DCD grafts in VITTAL trial (30% in DCD compared with 8.3% in DBD and 18% in comparative cohort) raised our concerns on NMP’s benefits for high-risk DCD grafts^7^. In our all-donor-typed analysis, NMP significantly reduced NAS and EAD, but these effects did not maintain in subgroup analysis. NMP only reduced major complications in ECD and EAD in DCD (Figs 4, 5), without exerting improvements in DBD subgroup and DBD&DCD subgroup [Figs S6 (Supplemental Digital Content 3, http://links.lww.com/JS9/A885) and S7 (Supplemental Digital Content 3, http://links.lww.com/JS9/A885)]. No benefits on graft/patient survival were observed in DBD, DCD, and ECD by NMP. Unlike HMP’s and NRP’s multiple benefits on OLT outcomes compared with SCS, our pooled data suggested that NMP might not provide optimal protection against IRI, especially for DCD and ECD grafts after a period of cold storage. In a recent study reporting 78 NMP-livers followed-up for 7 years by Hefler *et al*.^44^, no differences in long-term survival, major complications or biliary complications were observed (*P*>0.05). Even in ischemia-free OLT, continuous NMP ‘without any ischemia’ still failed to improve survival^43^. These results indicated that only NMP alone might be not enough for high-risk grafts. Though allowing ex-situ viability assessment at 37°C, NMP may still be associated with continuous warm ischemia of donor liver because the organ is metabolically active and current technology may not achieve sufficient hepatic perfusion compared with that in-situ. Therefore, this may partly explain the relatively high rates of NAS in NMP-DCD livers and limited benefits on survival^50^. Besides potential insufficient perfusion, user or device error might cause serious consequences in NMP, leading to graft loss. For this reason, it has been proposed that NMP should best be preceded by a short period of HMP as sequential HMP&NMP for high-risk DCD livers indeed demonstrated excellent results and low NAS risk^51^. Following HMP, a longer preservation time will allow NMP to modulate the quality of marginal donor by delivering various drugs, anti-oxidants, and siRNA, providing the possibility for organ repair, immune regulation, and even ectopic regeneration. Combined HMP and NMP strategy will be a very meaningful and promising attempt. More studies are warranted to further clarify NMP’s selection criteria for ECD and expand the boundary of this promising field.

NRP has considerable merits especially in countries with non-touching period, which may result in long warm ischemic injury^52^. Our data showed that NRP remarkably reduced the risk of PNF, NAS, EAD, and HAT in cDCD grafts. Surprisingly, our pooled data suggested that NRP could remarkably reduce PNF and HAT risk in NRP-cDCD compared with SCS-cDCD (Fig. 6). Naturally, this advantage also translates to significant improvements in 1-year graft/patient survival of NRP-cDCD (Fig. 7). NRP could also improve the outcomes of NRP-cDCD to a non-inferior level compared with SCS-DBD. Therefore, NRP could be considered as first choice for graft preservation in countries requiring long non-touch time as it both reduces discarded rates and improves graft survival.

Although using uDCD greatly expands the donor pool, its use in high-risk patients needs to be more cautious, and the protocols of NRP in uDCD-OLT should be more stringent. In contrast to cDCD, 1-year graft and patient survival in NRP-uDCD is still inferior to SCS-DBD (OR 0.44, *P*<0.01), suggesting that there is some kind of upper limit to NRP’s benefits under long time warm ischemia. Patrono *et al*.^53^ demonstrated that a combination of NRP and HMP for cDCD achieves comparable outcomes to standard SCS-DBD. This perfusion strategy has the potential to leverage the advantages of both NRP and HMP, while simultaneously addressing their respective limitations to optimize the prognosis of uDCD-OLT^53^. However, further multicenter RCTs are needed to validate the benefits of sequential perfusion strategies combining NRP with HMP or NMP.

In future, appropriate perfusion methods are required to be clarified to maximize clinical benefits for corresponding donor types. Theoretically, there could be an optimum perfusion strategy for a specific type of donors and even for each donor. Currently in the early periods of MP application, preliminary clinical trials were mainly designed to compared the efficacy and feasibility of different MP approaches with SCS as the clinical standard. A direct comparison between different MP techniques is still lacking, especially for current two main paradigms – NMP versus HOPE. Our results suggested the superiority of HMP on DCD and ECD. But owing to insufficient data of NMP-ECD studies, the superiority of HMP to NMP needs to be verified. Recently, the first multicenter RCT to test the effects of NMP versus HOPE on ECD-DBD livers is on recruitment and will help to answer this academic problem (*ClinicalTrials.gov: NCT04644744*).

According to our results, we currently recommend NRP and HMP as preferred treatment for ECD as they demonstrated significant improvements in OLT outcomes while NMP failed to achieve improvements in several critical endpoints. However, HMP is confronted with the challenge of testing graft viability. As a result, several research groups have recently attempted combinations of HMP and NMP. For critical high-risk donor livers initially declined if using SCS, recent studies have shown that pre-transplant sequential use of HMP&NMP enabled successful resuscitation and transplantation of these marginal grafts, which therefore increased the number of transplantable livers by 20%^51^. Sequential NRP and HMP also allows achieving comparable outcomes to standard DBD grafts in DCD grafts with prolonged WIT and high donor risk index^53^. Combined perfusion strategy seems to integrate the unique clinical benefits of NMP, HMP, and NRP, which could further enhance the rescue benefits by minimizing IRI and ultimately resuscitate the marginal grafts. It allows for more comprehensive and personalized preservation and assessment of organs, ultimately enhancing the utilization and successful transplantation of high-risk donors. However, this direction requires further investigation. Best application scenarios for different donors also needs to be investigated by further researches.

### Limitations

The present study has several limitations. First, clinical heterogeneity among different included studies might still exist though strict enrollment criteria were adopted during screening. Due to different study design, donor types, clinical characteristics, perfusion parameters, and perioperative management among different studies may result in analytical bias. Second, the sample size limitation for meta-analyses is unavoidable as MP technique has not been widely used in clinical and many large comparative trials are still in progress. Therefore, it might lack adequate power to identify differences in very low-risk complications such as NAS and graft loss. Third, the endpoints and follow-up in different studies are inconsistent. This could lead to attritions in valid data and thus no sufficient evidence can be obtained for certain endpoints. Fourth, lack of RCTs may result in over-estimating the benefits from small sample studies. Head-to-head RCTs directly comparing different perfusion strategies could assume paramount significance and provide direct insight into the priority of certain MP strategy. Considering these limitations, further large sample RCTs are needed to further confirm the benefits of different MP strategies on different graft types.

## Conclusions

In conclusion, this is the first comprehensive study analyzing the effects of different MP strategies on different donor types. Our study demonstrated that HMP could comprehensively improve OLT outcomes from all donor types, with strongest effects seen in ECD-OLT. Therefore, HMP has potential to be the first-lined choice for high-risk livers. NMP has some certain short-term outcomes but shows limited improvements in survival. Further studies for NMP-DCD and NMP-ECD are warranted. As in-situ perfusion technique, NRP has obvious short-term and long-term benefits for cDCD. NRP is recommended in countries with long non-touching period. These results may provide valuable reference for decision-making and future clinical trial design. Further investigations are warranted to determine the best perfusion strategy for different grafts types to maximize the OLT outcomes, especially for high-risk donors.

## Ethical approval

Not relevant.

## Consent

Not relevant.

## Sources of funding

This work was co-supported by the Beijing iGandan Foundation (RGGJJ-2021-008), the National Key R&D Program of China (2022YFA1104900), the National Natural Science Foundation of China (82200702 and 31972926), and the Guangdong Basic and Applied Basic Research Foundation (2020A1515111111).

## Author contribution

K.Z., Y.N., A.L.: study concept and design; A.L., Y.N., W.C., S.C., and L.Z.: acquisition of data; A.L., W.C., and P.C.: analysis and interpretation of data; A.L., Y.N., P.C.: drafting of the manuscript; Y.N. and K.Z.: critical revision of the manuscript for important intellectual content; Y.N. and K.Z.: administrative, technical, or material support; and K.Z.: study supervision. All authors have made a significant contribution to this study and have approved the final manuscript.

## Conflicts of interest disclosure

The authors declare that they have no conflicts of interest.

## Research registration unique identifying number (UIN)

The review protocol was registered prospectively with the National Institute for Health Research (PROSPERO) system and is available online with registration number CRD42023391864.

## Guarantor

Kebo Zhong and Yu Nie.

## Data availability statement

The data, code, and other materials are available from the corresponding author upon reasonable request. The data that support the findings of this study are openly available in PubMed.

## Provenance and peer review

Not commissioned, externally peer-reviewed.

## Supplementary Material
