# Supplementary material for: Effects of machine perfusion strategies on different donor types in liver transplantation: a systematic review and meta-analysis
Source: Int J Surg. 2023 Aug 11;109(11):3617–30. doi: 10.1097/JS9.0000000000000661 (PMC10651255; doi:10.1097/JS9.0000000000000661)
Supplement: Supplementary file 3 [file js9-109-3617-s003.docx]

Supplementary Information

Table of Contents

[Description of the distribution of baseline characteristics 2](#_Toc139900142)

[Risk of Bias 2](#_Toc139900143)

[Characteristics of NMP, HMP and NRP clinical studies 5](#_Toc139900144)

[Summary of machine perfusion strategies of NMP, HMP and NRP studies 9](#_Toc139900145)

[Definition of outcomes 17](#_Toc139900146)

[Figure legends 19](#_Toc139900147)

# Description of the distribution of baseline characteristics

39 studies were eligible in the systematic review, including 9 RCTs, and 30 cohort studies. A total of 6254 liver allograft recipients were included: 3970 SCS (control group), 608 grafts with hypothermic machine perfusion, 709 with normothermic machine perfusion and 968 with normothermic regional perfusion, of which 1122 livers were from RCTs.

In HMP group, 9 studies evaluated DBD grafts, 4 studies evaluated DCD grafts, 1 study evaluated a mix of both DBD and DCD livers. Among these, 11 studies evaluated ECD grafts. The median value of HMP median cold ischemia time and median functional time in included studies was 373 (311- 525) min, and 31 (23- 44.3) min, respectively. The median HMP perfusion time was 2.2 hours, ranged from 2 to 2.42 hours. The median value of median recipient Meld score was 15.2 (13.0-18.0).

In NMP group, 6 studies evaluated DBD grafts, 4 studies provided DCD results with only one studies investigating DCD, 9 studies evaluated a mix of both DBD and DCD livers. Among these, 5 studies evaluated ECD grafts. The median value of NMP median cold ischemia time and median functional time in included studies was 360 (235.5- 411.5) min and 22 (21.0- 30.6) min, respectively. The median value of median NMP perfusion time was 7.7 hours, ranged from 4.7 to 9.1 hours. The median value of median recipient Meld score was 15(13.3-19.1).

In NRP group, 7 studies evaluated controlled DCD grafts and 3 studies evaluated uncontrolled DCD grafts. Among these, 3 studies used DCD as control group while 7 studies used DBD as control group. The median value of NRP median cold ischemia time and median functional time in included studies was 345 (313.3- 372) min and 19 (12- 29) min, respectively. The median HMP perfusion time was 2.02 hours, ranged from 1.9 to 3.2 hours. The median value of median recipient Meld score was 12 (11.3-14.3).

# Risk of Bias

**Table S1**. Risk of bias assessment of included nonrandomized studies using the ROBINS-I tool

| Study | confounding | Selection of participants | Classification of interventions | Deviations from intended interventions | Missing data | Measurement of outcomes | Selection of the reported result | Overall |
| --- | --- | --- | --- | --- | --- | --- | --- | --- |
| NMP |  |  |  |  |  |  |  |  |
| Jassem, W et al.2019 | Moderate | Serious | Low | Low | Low | Low | Low | Serious |
| Fodor, M. et al.2021 | Moderate | Low | Low | Low | Low | Low | Low | Moderate |
| Hann, A. et al.2022 | Moderate | Low | Low | Low | Low | Low | Low | Moderate |
| Selzner, M. et al.2016 | Serious | Low | Low | Serious | Low | Low | Low | Serious |
| Ravikumar, R. et al. 2016 | Serious | Low | Low | Low | Low | Low | Low | Serious |
| Mergental H. et al. 2020 | Moderate | Moderate | Low | Low | Low | Low | Low | Moderate |
| Liu, Q. et al.2020 | Moderate | Low | Low | Serious | Low | Low | Low | Serious |
| Gaurav, R. et al.2022 | Moderate | Low | Low | Low | Low | Low | Low | Moderate |
| Watson, C. J. E. et al.2017 | Serious | Moderate | Low | Serious | Low | Low | Low | Serious |
| Guo, Z.Y. et al. 2021 | Moderate | Moderate | Low | Low | Low | Low | Low | Moderate |
| Hefler, J. et al. 2023 | Moderate | Low | Low | Low | Low | Low | Low | Moderate |
| HMP |  |  |  |  |  |  |  |  |
| Guarrera, J. V. et al. 2010 | Moderate | Low | Low | Low | Low | Low | Low | Moderate |
| Guarrera, J. V. et al. 2015 | Moderate | Low | Low | Low | Low | Low | Low | Moderate |
| Schlegel, A. et al.2019 | Moderate | Low | Low | Moderate | Low | Low | Low | Moderate |
| Rayar, M. et al.2021 | Moderate | Moderate | Low | Low | Low | Moderate | Low | Moderate |
| van Rijn, R. et al.2017 | Serious | Serious | Low | Low | Low | Low | Low | Serious |
| Patrono, D. et al.2019 | Moderate | Moderate | Low | Low | Low | Low | Low | Moderate |
| Dutkowski, P. et al. 2015 | Moderate | Low | Low | Low | Low | Low | Low | Moderate |
| Ravaioli, M. et al.2020 | Moderate | Low | Low | Low | Low | Low | Low | Moderate |
| Patrono.D. et al.2022 | Moderate | Low | Low | Low | Low | Low | Low | Moderate |
| Horné, F. et al.2022 | Serious | Low | Low | Low | Low | Low | Low | Serious |
| NRP |  |  |  |  |  |  |  |  |
| Gaurav, R.2022 | Moderate | Low | Low | Low | Low | Low | Low | Moderate |
| Hessheimer, A. J.2021 | Moderate | Low | Low | Low | Low | Low | Low | Moderate |
| Miñambres, E.2020 | Serious | Low | Low | Moderate | Low | Low | Low | Serious |
| Rodriguez, R. P. et al.2022 | Serious | Low | Low | Serious | Low | Low | Low | Serious |
| Savier, E. et al.2015 | Moderate | Low | Low | Low | Low | Low | Low | Moderate |
| Fondevila et al.2012 | Moderate | Low | Low | Low | Low | Low | Low | Moderate |
| Romero, J.C. et al.2019 | Moderate | Low | Low | Low | Low | Low | Low | Moderate |
| Savier, E. et al.2020 | Moderate | Moderate | Low | Low | Low | Low | Low | Moderate |
| Schurink, I. J. et al. 2022 | Moderate | Low | Low | Low | Low | Low | Low | Moderate |
| Patricia, R. et al.2021 | Moderate | Low | Low | Low | Low | Low | Low | Moderate |

**Table S2**. Risk of bias assessment of included randomized studies using the RoB2 tool

| Study | Randomization process | Deviations from intended interventions | Missing outcome data | Measurement of the outcome | Selection of the reported result | Overall |
| --- | --- | --- | --- | --- | --- | --- |
| NMP |  |  |  |  |  |  |
| Ghinolfi, D et al. 2019 | Low | Low | Low | Low | Low | Low |
| Nasralla, D. et al. 2018 | Low | Low | Low | Low | Low | Low |
| Markmann, J. F. et al. 2022 | Low | Low | Low | Low | Low | Low |
| Minor, T et al.2022 | Low | Low | Low | Low | Low | Low |
| Guo, Z.Y. et al. 2023 | Low | Low | Low | Low | Low | Low |
| HMP |  |  |  |  |  |  |
| van Rijn, R. et al. 2021 | Low | Low | Low | Low | Low | Low |
| Czigany, Z. et al.2021 | Low | Low | Low | Low | Low | Low |
| Schlegel, A. et al. 2023 | Low | Low | Low | Low | Low | Low |
| Ravaioli, M et al. 2022 | Low | Low | Low | Low | Low | Low |

# Characteristics of NMP, HMP and NRP clinical studies

**Table S3**. Characteristics of clinical NMP studies

| Study | Study type | Study location | Group | No. DBD or DCD grafts | Donor age  (years) | Recipient age  (years) | Recipient MELD(mean) or Child Score |
| --- | --- | --- | --- | --- | --- | --- | --- |
| Ghinolfi, D et al. 2019 | RCT | Italy;  1 center | NMP | ECD: 10DBD | 81 (77.5–87.2) | 57 (46–61) | 12.5 (9–16) |
|  |  |  | SCS | ECD: 10DBD | 80 (72–87.2) | 55 (43–61) | 9.5 (8–15) |
| Nasralla, D. et al. 2018 | RCT | Europe;  7 centers | NMP | 87DBD,34DCD | 56 (45–67) | 55 (48–62) | 13 (10–18) |
|  |  |  | SCS | 80DBD,21DCD | 56 (47–66) | 55 (48–62) | 14 (9–18) |
| Markmann, J. F. et al. 2022 | RCT | UK;  20 centers | NMP | 124DBD;28DCD | 47.5 (10.9–83.7) | 59.2 (19.5–76.6) | 29.0 (6.0–49.0) |
|  |  |  | SCS | 133DBD;13DCD | 45.8 (13.0–80.6) | 61.4 (20.8–77.8) | 29.0 (9.0–46.0) |
| Minor, T et al.2022 | RCT | Germany;  1 center | NMP | 20ECD | 63.65 ± 12.75 | 57.50 ± 6.97 | 16.20 ± 8.30 |
|  |  |  | SCS | 20ECD | 63.50 ± 16.18 | 48.65 ± 14.47 | 18.70 ± 8.12 |
| Guo, Z.Y. et al. 2023 | RCT | China;  1 center | NMP | 32DBD | 47 (39-55) | 53 (46-62) | 15 (11-21) |
|  |  |  | SCS | 33DBD | 43 (33-49) | 54 (44-58) | 16 (11-24) |
| Jassem, W et al. 2019 | Cohort study | UK;  1 center | NMP | 12DBD | 60 (41 – 85) | 54 (38 – 62) | 15 (9 – 27) |
|  |  |  | SCS | 27DBD | 54 (26 – 82) | 50 (17 – 70) | 16 (2 – 26) |
| Fodor, M. et al. 2021 | Cohort study | Austria;  1 center | NMP | 50DBD;9DCD | 57.0 (22.0) | 63.4 (12.7) | 15 of 58 (12) |
|  |  |  | SCS | 55DBD;4DCD | 56.0 (16.5) | 60.6 (10.5) | 17 (10) |
| Hann, A. et al. 2022 | Cohort study | UK;1 center | NMP | 26DBD | 51 (40–63) | 39 (26–52) | 21 (13–25) |
|  |  |  | SCS | 25DBD | 49 (44–63) | 50 (31–58) | 19 (15–25) |
| Selzner, M. et al. 2016 | Cohort study | Canada;  1 center | NMP | ECD: 8DBD 2DCD | 48 (17–75) | 56 (45–71) | 21 (8–40) |
|  |  |  | SCS | ECD: 24DBD 6DCD | 46 (22–68) | 54 (42–63) | 23 (7–37) |
| Ravikumar, R. et al. 2016 | Cohort study | UK;  2 centers | NMP | 16DBD,4DCD | 58.0(21–85) | 54.4(33–66) | 12(7–27) |
|  |  |  | SCS | 22DBD,8DCD | 58.5(21–82) | 55.0(27–65) | 14(6–25) |
| Mergental H. et al. 2020 | Cohort study | UK;  1 center | NMP | 12DBD,10DCD | 56(45–65) | 56(46–65) | 12(9–16) |
|  |  |  | SCS | 24DBD,20DCD | NA | NA | NA |
| Liu, Q. et al. 2020 | Cohort study | USA;  1 center | NMP | 13DBD,8DCD | 35.0 ± 12.7 | 57.0 ± 7.1 | 19.1 ± 7.7 |
|  |  |  | SCS | 52DBD,32DCD | 34.8 ± 15.0 | 57.4 ± 8.4 | 19.4 ± 8.7 |
| Gaurav, R. et al. 2022 | Cohort study | UK;  1 center | NMP | ECD: 67DCD | 52 (29–60) | 59 (51–63) | 14 (10–18) ** |
|  |  |  | SCS | ECD: 97DCD | 50 (36–59) | 56 (50–62) | 16 (13–20) ** |
| Watson, C. J. E. et al. 2017 | Cohort study | UK;  1 center | NMP | ECD:3DBD;9DCD | 56 (24–67) | 57 (46–65) | 17 (10–26) |
|  |  |  | SCS | ECD:6DBD;18DCD | 54 (22–72) | NA | 1.8 (1.1–3.3) |
| Guo, Z.Y. et al. 2021 | Cohort study | China;  1 center | NMP | 38 DBD | 36.0 ± 14.2 | 50.8 ± 11.3 | 24.0 ± 3.7 |
|  |  |  | SCS | 130DBD | 37.2 ± 12.2 | 50.2 ± 9.6 | 24.0 ± 4.1 |
| Hefler, J. et al. 2023 | Cohort study | Canada;  1 center | NMP | 63DBD;16DCD | 40 (25-56) | 56.0 (45.7-62.4) | 19 (13-27) |
|  |  |  | SCS | 335DBD;51DCD | 37 (26-53) | 58.1 (46.9-63.1) | 16 (11-23) |

Data is presented as mean ± standard deviation or median (range), *P＜0.05，**P＜0.01，***P＜0.001. SCS, static cold storage; ECD, extended/expanded criteria donor; DCD, donation after circulatory death; DBD, donation after brain death; NMP, normothermic machine perfusion; MELD, model for end-stage liver disease;

**Table S4**. Characteristics of clinical HMP studies

| Study | Study type | Study location | Group | No.DBD or DCD grafts | Donor age | Recipient age | Recipient MELD(mean) or Child Score |
| --- | --- | --- | --- | --- | --- | --- | --- |
| van Rijn, R. et al. 2021 | RCT | Switzerland;2 centers | HMP | ECD: 78DCD | 52(43–57) | 60(52–65) | 14(10–19) |
|  |  |  | SCS | ECD: 78DCD | 49(37–59) | 60(52–65) | 16(10–22) |
| Guarrera, J. V. et al. 2010 | Cohort study | USA;  1 center | HMP | 20DBD | 39.4 ± 2.5 | 55.4 ± 6.2 | 17.2 ± 7.4 |
|  |  |  | SCS | 20DBD | 45.6 ± 2.1 | 52.7 ± 8.9 | 16.8 ± 6.8 |
| Guarrera, J. V. et al. 2015 | Cohort study | USA;  1 center | HMP | 31ECD | 57.5 ± 17.8 | 57.5 ± 8.0 | 19.5 ± 5.9 |
|  |  |  | SCS | 30ECD | 57.9 ± 16.9 | 58.4 ± 9.6 | 21.4 ± 6.3 |
| Schlegel, A. et al. 2019 | Cohort study | Switzerland;2 centers | HMP | ECD: 50DCD | 57(47–67)* | 58(56–62) | 11(8–14) |
|  |  |  | SCS | ECD: 50DCD | 53(33–60)* | 57(51–61) | 11.8(8.5–15.8) |
| Rayar, M. et al. 2021 | Cohort study | France;  1 center | HMP | ECD: 25DBD | 70 (45–87) | 63 (43–69) | 18.3 (7–37) |
|  |  |  | SCS | ECD: 69DBD | 72 (25–88) | 62 (36–70) | 18.3 (5–40) |
| van Rijn, R. et al. 2017 | Cohort study | Netherlands;  1 center | HMP | ECD: 10DCD | 53 (47–57) | 57 (54–62) | 16 (15–22) |
|  |  |  | SCS | 20DCD | 53 (47–58) | 52 (42–60) | 22 (17–27) |
| Patrono, D. et al. 2019 | Cohort study | Italy;  1 center | HMP | ECD: 25DBD | 74.3 ± 10.9 | 56.3 ± 9 | 15.3 ± 8.6 |
|  |  |  | SCS | ECD: 50DBD | 74.9 ± 10.3 | 55.9 ± 7.4 | 15.5 ± 8.5 |
| Dutkowski, P. et al. 2015 | Cohort study | Europe;  3 centers | HMP | ECD: 25DCD | 54 (36–63) | 60 (57–64) *** | 13 (9–15) |
|  |  |  | SCS | ECD: 50DCD | 48 (33–51) | 56 (49–59) *** | 16(10–21) |
| Ravaioli, M. et al. 2020 | Cohort study | Italy;  1 center | HMP | ECD: 10DBD | 77.5(60–84) | 57.5(50–68) | 13(7–16) |
|  |  |  | SCS | ECD: 30DBD | 75.5(53–85) | 60.5(48–68) | 13.5(7–20) |
| Czigany,Z. et al.2021 | RCT | Europe;  4 centers | HMP | ECD: 23DBD | 73 (60–78) | 60 (52–64) | 13 (9–18) |
|  |  |  | SCS | ECD: 23DBD | 71 (59–78) | 63 (56–67) | 17 (8–25) |
| Patrono.D. et al.2022 | Cohort study | Italy;  1 center | HMP | ECD: 121DBD | 75.7(62.5– 82.7) * | 60.5 (55.5– 65.0) | 13.0 (9.0– 17.0)* |
|  |  |  | SCS | ECD: 723DBD | 74.3(66.2– 78.8) * | 59.8 (55.3– 63.6) | 12.0 (9.0– 17.0)* |
| Horné, F. et al. 2022 | Cohort study | Germany;  1 center | HMP | 50DBD | 49.2(44.4–54.0) * | 53.0 (50.3–55.8) | 22.1 (19.2–25.1) |
|  |  |  | SCS | 50DBD | 55.1(50.5–59.6) * | 50.6 (47.2–54.0) | 20.4 (17.6–23.2) |
| Schlegel, A. et al. 2023 | RCT | Europe;  10 centers | HMP | 85DBD | 62.0 (44.0–71.0) | 60.0 (51.0– 64.0) | 20.0 (11.0– 28.0) |
|  |  |  | SCS | 85DBD | 62.0 (44.0–71.0) | 57.0 (49.0– 64.0) | 19.0 (12.0– 26.0) |
| Ravaioli, M et al.  2022 | RCT | Italy | HMP | ECD: 55 DBD | 76 (64-81) | 57(47-65) | 15(10-18) |
|  |  | 1 center | SCS | ECD: 55 DBD | 72 (59-77) | 60(53-66) | 14(9-20) |

Data is presented as mean ± standard deviation or median (range), *P＜0.05，**P＜0.01，***P＜0.001. SCS, static cold storage; ECD, extended/expanded criteria donor; DCD, donation after circulatory death; DBD, donation after brain death;, HMP, hypothermic machine perfusion; MELD, model for end-stage liver disease

**Table 5**. Characteristics of clinical NRP studies

| Study | | study type | study location | Group | No.DBD or DCD grafts | Donor age | Recipient age | Recipient MELD (mean) or Child Score |
| --- | --- | --- | --- | --- | --- | --- | --- | --- |
| Gaurav, R. 2022 | | Cohort study | UK;1 center | NRP | 69cDCD | 51 (33–58) | 56 (48–63) | 14 (10–16) ** |
|  |  |  |  | SCS | 97cDCD | 50 (36–59) | 56 (50–62) | 16 (13–20) ** |
| Hessheimer, A. J. 2021 | | Cohort study | Spain;22 centers | NRP | 545cDCD | 59 (49– 67) | 59 (53– 63) | 12 (9– 17 ) |
|  |  |  |  | SCS | 258cDCD | 58 (48– 67) | 58 (53– 63) | 12 (8– 16) |
| Miñambres, E. 2020 | | Cohort study | Spain;2 centers | NRP | 16cDCD | 54 (47‐59) | 60 (52‐64) | 12 (8‐21) |
|  |  |  |  | SCS | 29DBD | 62 (52‐67) | 60 (53‐64) | 15 (13‐18) |
| Rodriguez, R. P. et al. 2022 | | Cohort study | Spain;1 center | NRP | 39cDCD | 52 (15–68)** | 54.49 (35–70) | 14.48 (6–26) |
|  |  |  |  | SCS | 78DBD | 59.4 (25–81)** | 56.44 (16–71) | 16.14 (6–27) |
| Savier, E. et al. 2015 | | Cohort study | France;3 centers | NRP | 13uDCD | 37 ± 3 | 54 ± 4 | 8.8 ± 2.5 |
|  |  |  |  | SCS | 41DBD | 55 ± 3 | NA | NA |
| Fondevila et al. 2012 | Cohort study | | Spain;1 center | NRP | 43uDCD | 47 (27–56) | 55 (49–60) | 19 14–21) |
|  |  |  |  | SCS | 538DBD | NA | NA | NA |
| Romero, J.C. et al. 2019 | | Cohort study | Spain; 1 center | NRP | 75uDCD | 41.7 ± 9.7*** | 58.8 ± 7.7*** | 14.4 ± 4.9 |
|  |  |  |  | SCS | 265DBD | 47.8 ± 14.9*** | 54.7 ± 10.1*** | 14.5 ± 6.3 |
| Savier, E. et al. 2020 | | Cohort study | French;6 center | NRP | 50cDCD | 50.0 (39.0–56.5) | 59.9 (54.1–63.9) | 7 (6–12) |
|  |  |  |  | SCS | 100DBD | 50 (40.0–59.0) | 58.4 (52.8–62.2) | 10 (6–14) |
| Schurink, I. J. et al. 2022 | | Cohort study | Netherlands; 1 center | NRP | 20cDCD | 67 (64–71) *** | 60 (52–64) | 11 (8–15) |
|  |  |  |  | SCS | 49cDCD | 48 (34–55) *** | 60 (51–66) | 14 (9–19) |
| Patricia, R. et al. 2021 | | Cohort study | Spain; 1 center | NRP | 100cDCD | 62 (53– 69) | 59 (54– 64) | 12 (9– 18) |
|  |  |  |  | SCS | 200DBD | 62 (50.8– 72) | 58 (53– 64) | 12 (9– 16) |

Data is presented as mean ± standard deviation or median (range), *P＜0.05，**P＜0.01，***P＜0.001. SCS, static cold storage; ECD, extended/expanded criteria donor; cDCD, controlled donation after circulatory death; uDCD, uncontrolled donation after circulatory death; DBD, donation after brain death; NRP, normothermic regional perfusion; MELD, model for end-stage liver disease

# Summary of machine perfusion strategies of NMP, HMP and NRP studies

**Table S6**. Summary of the machine perfusion strategies of NMP studies

| Study | Group | CIT（min） | fWIT（min） | Total perservation time (h) | Perfusion duration (h) | Perfusion settings |
| --- | --- | --- | --- | --- | --- | --- |
| Ghinolfi, D et al. 2019 | NMP | 280 (242–297)*** | 74 (70–82) | NA | 4.2 (3.3–4.7) | PV+HA  blood-based perfusate  LiverAssist  37℃  4L/min 30% O2 flow (PaO2 200-250mmHg)  Terminal perfusate pH ranged from 7.19 to pH: 7.44 |
|  | SCS | 394 (366–465)*** | 69 (62–78) | NA |  |  |
| Nasralla, D. et al. 2018 | NMP | 126(106.5–143.0) | 21 (17–25)** | 11.9 (9.0–14.6)*** | 9.1 (6.2–11.8) | PV+HA  3 units packed RBCs + 0.5L Gelofusine  OrganOx metra  37℃  physiological PaO2  (7.31 ± 0.17)  HA:65–75 mm,inferior vena cava pressure (0–2 mm Hg) |
|  | SCS | 465(375-575) | 16 (10–20)** | 7.8 (6.3–9.6)*** |  |  |
| Jassem, W et al. 2019 | NMP | NA | NA | NA | NA | PV+HA  3 units packed RBCs+1 unit Gelofusine  OrganOx metra  37℃  PaO2 ~12kPa  7.2-7.4 |
|  | SCS | 571 ±160 | NA | NA |  |  |
| Fodor, M. et al. 2021 | NMP | 360 (120) | NA | 21 (12)*** | NA | OrganOx metra |
|  | SCS | 420 (180) | NA | 7 (3)*** |  |  |
| Hann, A. et al. 2022 | NMP | 364 (181–575) *** | NA | 18.5 (12.4–22.1) *** | 12.1 (7.9–15.6) | PV+HA  3 units O-negative red blood cells+0.5LGelofusine  OrganOx metra  physiological temperatures |
|  | SCS | 465 (409–635) *** | NA | 8.2 (6.5–9.7) *** |  |  |
| Selzner, M. et al. 2016 | NMP | Not available | 49 (21–76) | 9.8 (3.7–12.2) | 8 (5.7–9.7) | PV+HA  3 units packed red blood cells+0.5LSteen solution  Metra device  37℃  PO2~14.4mm Hg（12.8-14.4mm Hg）  7.3-7.5 |
|  | SCS | 634(523-783) | 46 (39–67) | 10.6 (8.7–13.1) |  |  |
| Ravikumar, R. et al. 2016 | NMP | NA | 21(14–31) | 9.3(3.5–18.5) | NA | PV+HA  3 units packed RBCs+1 unit Gelofusine  OrganOx metra  37℃  PaO2 ~12kPa  7.2-7.4 |
|  | SCS | 534(252–684) | 15(9–23) | 8.9(4.2–11.4) |  |  |
| Mergental H. et al. 2020 | NMP | 452 (389–600) | 22.5(19.0–35.0) | 17.9 (16.3–21.8) | 9.3(3.5–18.5) | PV+HA  UW  OrganOx metra  active oxygenation |
|  | SCS | NA | NA | NA |  |  |
| Liu, Q. et al. 2020 | NMP | 191 ± 45 | 21 ± 5 | 8.8 ± 1.1 | 9.8 (7.5–11.8) | PV+HA  4 units FFP + 4units PRBCs + 200mL 25% albumin  Non-commercial, institutional perfusion device  36℃  active oxygenation  7.15 ± 0.15→7.51 ± 0.11 |
|  | SCS | 498 ± 90 | NA | 8.3 ± 1.5 |  |  |
| Gaurav, R. et al. 2022 | NMP | 396 (346–441) *** | 15 (12–18) *** | 15.7(13.6–17.5) *** | 4.9 (3.4–7.9) | PV+HA  UW  Liver Assist or OrganOx metra |
|  | SCS | 430 (397–474) *** | 15 (11–18) *** | 7.2(6.6–7.9) *** |  |  |
| Watson, C. J. E. et al. 2017 | NMP | 427 (222–877) | 31 (17–160) | NA | 7.7（5.5–9.5） | PV+HA  3 units of leucocyte-depleted washed red cells+ a liter of either succinylated gelatin or Steen solution  Liver Assist  37°C  pO2~20 kPa (153 mm Hg),SO2~98% to 99%  HA:60mmHg;PV:9mmHg |
|  | SCS | 439 (333–720) | 22 (12–124) | NA |  |  |
| Markmann, J. F. et al. 2022 | NMP | 175.4（43.5） | NA | NA | 4.7(2–8.8) | PV+HA  4 to 5 units of packed red blood cells + 4% amino acids and 10% dextrose  Organ Care System  34°C  PaO2 (mmHg)~420.2 ± 80.7  7.43 ± 0.1  HA:70.6 ± 16.2; PV:5.4 ± 2.3 |
|  | SCS | 338.8（91.5) | NA | NA |  |  |
| Minor, T et al.2022 | NMP | 485 ± 82 | 29.5 ± 5.3 | NA | 4.6 ± 2 | PV+HA  HTK/UW,  Liver Assist,  20°C  active oxygenation  4 mmHg at the portal vein and 25 mmHg (60 bpm) at the hepatic artery |
|  | SCS | 454 ± 83 | 27.1 ± 6.3 | NA |  |  |
| Guo, Z.Y. et al. 2023 | NMP  SCS | NA  414 (396-438) | NA  NA | 7.1 (6.7-7.6)  NA | 7.1 (6.7-7.6)  NA | PV+HA  leucocyte-depleted red blood cells-based perfusate  Liver Assist  37°C |
| Guo, Z.Y. et al. 2021 | NMP  SCS | 0***  369 (329-450) *** | NA  NA | 4 (2.7-6)  NA | 4 (2.7-6)  NA | PV+HA  leucocyte-depleted red blood cells-based perfusate  Liver Assist  37°C |
| Hefler, J. et al. 2023 | NMP  SCS | 359 (301-412) ***  288.5 (193-383) *** | 21.5(15-25.5)19 (12-27) | 14.1(12.8-16.1) ***  4.8(3.2-6.4) *** | 7.9 (6.45-10.33) | PV+HA  3 units packed RBCs + 0.5L Gelofusine  OrganOx metra  7.35-7.45 |

Data is presented as mean ± standard deviation or median (range), *P＜0.05，**P＜0.01，***P＜0.001. CIT, cold ischaemic times; fWIT, function warm ischemic time; SCS, static cold storage;

NMP, normothermic machine perfusion; PV: portal vein; HA, Hepatic artery

**Table S7**. Summary of the machine perfusion strategies for HMP studies

| Study | Group | CIT（min） | fWIT（min） | Total perservation time (h) | Perfusion duration (h) | Perfusion settings |
| --- | --- | --- | --- | --- | --- | --- |
| van Rijn, R. et al. 2021 | HMP | 371 (316-415) *** | 29 (22-33) | 8.7 (7.8-9.3) | 2.2 (2-2.6) | PV+HA  4L Belze UW MPS  Liver Assist  10℃  500ml/ min 100% O2 flow  HA:25mm Hg;PV:5mm Hg |
|  | SCS | 409 (356-477) *** | 27(21-35) | 6.8 (5.9-8.0) |  |  |
| Guarrera, J. V. et al. 2010 | HMP | 564±126 | 44.3 ± 6.5 | NA | 4.3 ± 0.9 | PV+HA  Vasosol  4-8℃  no active oxygenation  HA: 5.5 ± 0.15 SEM mmH; PV; 2.9 ± 0.08 SEM mmHg |
|  | SCS | 534 ± 168 | 45.1 ± 6.7 | NA |  |  |
| Guarrera, J. V. et al. 2015 | HMP | 558 ± 96 | 45.6 ± 7.3* | NA | 3.8 ± 0.9 | PV+HA  Vasosol  4-8℃  no active oxygenation  HA: 5.1 mmHg 0.2 SEM;PV:2.9 mmHg 0.1SEM |
|  | SCS | 516 ± 144 | 40.0 ± 8.3* | NA |  |  |
| Schlegel, A. et al. 2019 | HMP | 264(210-312) | 31 (27-36) *** | 6.0 (5.0-7.0) | 2 (1.6-2.4) | PV perfusion only  3L Belzer UW MPS  Liver Assist  10-12ºC  PaO2 80-100kPa |
|  | SCS | 282(258-318) | 17 (15-19) *** | 4.7 (4.3-5.3) |  |  |
| Rayar, M. et al. 2021 | HMP | 525 (379-824) | NA | NA | 2.0 (1.3-4.2) | PV perfusion only  2L Belzer UW MPS  Liver Assist  11ºC  1L/min 100% O2 flow  PV:3-5 mmHg |
|  | SCS | 555 (207-722) | NA | NA |  |  |
| van Rijn, R. et al. 2017 | HMP | NA | 15 (13-17) | 8.7 (7.8-9.9) | 2.1 (2.1-2.3) | PV+HA  4L Belzer UW MPS  Liver Assist  10ºC  500ml/min 100% O2 flow (PaO2 >450mmHg)  HA:25mm Hg;PV:5mm Hg |
|  | SCS | 503(476-526) | 16 (14-18) | 8.4 (7.9-8.8) |  |  |
| Patrono, D. et al. 2019 | HMP | 311 ± 53*** | 23 ± 7 | 8.3 ± 1.0*** | 3.1 ± 0.8 | PV + HA perfusion (except for 2 cases PV perfusion only)  3L Belzer UW MPSior  Liver Assist  10ºC  PaO2 600mmHg  HA:25mm Hg;PV:5mm Hg |
|  | SCS | 391 ± 72*** | 24 ± 5 | 6.5 ± 1.2*** |  |  |
| Dutkowski, P. et al. 2015 | HMP | 188(141-264) *** | 31 (26-36) *** | 5.3 (4.7-6.5) ** | 2.0 (1.7-2.5) | PV perfusion only  UW gluconate solution (KPS-1)  Liver Assist  10℃  pO2 80-100kPa |
|  | SCS | 395(349-447) *** | 23 (20-29) *** | 6.6 (5.8-7.5) ** |  |  |
| Ravaioli, M. et al. 2020 | HMP | 426(366–576) | NA | NA | 2.2 (1–3.5) | PV perfusion only  Belzer UW MPS  Non–commercial, institutional perfusion device  4ºC  PaO2 600–750mmHg  5 mmHg |
|  | SCS | 420(324–600) | NA | NA |  |  |
| Czigany,Z. et al.2021 | HMP | 495 (447–612) | 39 (35–54) | NA | 2.42(1.68-3.38) | portal vein only  Belzer UW–MPS  Liver Assist  10℃  pO2 60–80 kPa |
|  | SCS | 502 (467–584) | 45 (39–52) | NA |  |  |
| Patrono.D. et al.2022 | HMP | 348 (318–399) | NA | 8.3(7.7–9) | 2.28 (1.87-3) | PV+HA  3 L Belzer MP  10℃ |
|  | SCS | 437 (387–489) | NA | 7.3(6.4–8.2) |  |  |
| Horné, F. et al. 2022 | HMP | 608.0 (566.5–649.6) | 52.4 (46.4–58.5) | NA | NA | portal vein only  UW–MPS  LiverAssist  8–12 ◦C  PV:3–5 mmHg |
|  | SCS | 599.0 (554.3–643.7) | 58.1 (52.0–64.2) | NA |  |  |
| Schlegel, A. et al. 2023 | HMP | 373.0 (299.2– 471.8) | NA | 7.9(6.73-9.8) | 1.59(1.22– 2.28) | portal vein only 3L re–circulating Belzer MPS Liver Assist  8–12 ◦C |
|  | SCS | 427.0 (356.0– 487.0) | NA | 7.12(5.93-8.12) |  |  |
| Ravaioli, M et al.  2022 | HMP | 255 (215–­325) | NA | 400 (360-480) | 2.4 (2­3.1) | portal vein only;  T: 10°C  Pressure: PV 3mmHg  Vitasmart  pH 7.02 (6.98 – 7.07) |
|  | SCS | 420 (360–­450) | NA | 420 (360­450) |  |  |

Data is presented as mean ± standard deviation or median (range), *P＜0.05，**P＜0.01，***P＜0.001. CIT, cold ischaemic times; fWIT, function warm ischemic time; SCS, static cold storage;

HMP, hypothermic machine perfusion; PV: portal vein; HA, Hepatic artery

**Table S7**. Summary of the machine perfusion strategies for NRP studies

| Study | Group | CIT（min） | fWIT（min） | perfusion duration (h) | Perfusion settings |
| --- | --- | --- | --- | --- | --- |
| Gaurav, R. 2022 | NRP | 399 (341–471) *** | 19 (15–24) *** | 2.22(2.02-2.38) | UW preservation solution |
|  | SCS | 430 (397–474) *** | 15 (11–18) *** |  |  |
| Hessheimer, A. J. 2021 | NRP | 320 (270– 379) | 12 (9– 16) *** | 1.85(1.35-2.10) | UW or IGL-1/Celsior/HTK/Other  35– 37ºC |
|  | SCS | 333 (284– 388) | 14 (11– 20) *** |  |  |
| Miñambres, E. 2020 | NRP | 311 ± 88 | 12 (10‐13) | 1.90(0.95-2.00) | femoral artery (FA) and femoral vein  37°C  pH: 7.35‐7.45  60-65 mm Hg in the FA cannula |
|  | SCS | 324 ± 113 | NA |  |  |
| Rodriguez, R. P. et al. 2022 | NRP | 298^a^ | 13.10 (6-26) | 0.82^a^ | NA |
|  | SCS | 310^a^ | NA |  |  |
| Savier, E. et al. 2015 | NRP | 348 ± 31 | 49 ± 4 | 4.00(3.94-4.30) | 32-33°C  pH: 7.0–7.4 |
|  | SCS | 456 ± 16 | 56 ± 4 |  |  |
| Fondevila et al. 2012 | NRP | 380 (325–430) | NA | 3.30(3.05-3.75) | UW  35.5-37.5°C |
|  | SCS | NA | NA |  |  |
| Romero, J.C. et al. 2019 | NRP | 386 ± 86 | 62 ± 14** | NA | Celsior; Belzer |
|  | SCS | 406 ± 163 | 70 ± 36** |  |  |
| Savier, E. et al. 2020 | NRP | 348 (300–402) * | 22 (20–26.8) | 3.17(2.52-3.72) | IGL-1/ SCOT 15/Celsior/Other  32-33°C  7.0–7.4 |
|  | SCS | 378 (324–438) * | NA |  |  |
| Schurink, I. J. et al. 2022 | NRP | 342 (294–387) | 29 (26–33) *** | 2.00(1.97-2.13) | Organ Assist/CardioHelp  100-200mm Hg  7.25-7.45 |
|  | SCS | 359 (300–392) | 24 (19–28) *** |  |  |
| Patricia, R. et al. 2021 | NRP | 274 (241– 311) | 10 (8.5– 12.2) | 2.02(1.97-2.13) | NA |
|  | SCS | 264 (228– 340) | NA |  |  |

Data is presented as mean ± standard deviation or median (IQR), ^a^ mean, *P＜0.05，**P＜0.01，***P＜0.001. CIT, cold ischaemic times; fWIT, function warm ischemic time; SCS, static cold storage; NRP, normothermic regional perfusion

# Definition of outcomes

EAD was defined according to Olthoff et al^1^.PNF is defined as liver failure without an identifiable cause that necessitated retransplantation or led to death within 7 days after transplantation^2^.Major complication defined as major complications defined by a Clavien Dindo score ≥3^3^, There is no consensus on the precise definition of NAS and different names and criteria have been used in the literature. Synonyms for NAS that are frequently used in the literature are ischemic–type biliary lesions and ischemic cholangiopathy^4^. Thus, some researchers propose to use the more general term post-transplant cholangiopath^5^. Definitions for sake of clarity, we elucidated the concepts of NAS and IC in different literature. NAS is most commonly defined as any irregularity or narrowing of the lumen of the intrahepatic or extrahepatic donor bile ducts assessed by MRCP at 6 months, excluding the biliary anastomosis ( van Rijn^2^, Mergental^6^, Gaurav^7^, van Rijn^8^). Although not the same, the other studies generally point to NAS. We classify the IC that was synonymous with NAS or simply represents intrahepatic strictures as NAS. PRS, defined as a decrease of more than 30% in the mean systemic arterial blood pressure within 10 minutes after reperfusion, with or without a doubling of the norepinephrine dose; primary nonfunction, defined as liver failure, without an identifiable cause, that necessitated retransplantation or led to death within 7 days after transplantation^2^. AKI was defined and graded according to 2012 KDIGO guidelines^9^, and we only counted moderate-to-severe AKI (2-3 stage).

1. Olthoff, K. M.; Kulik, L.; Samstein, B.; Kaminski, M.; Abecassis, M.; Emond, J.; Shaked, A.; Christie, J. D., Validation of a Current Definition of Early Allograft Dysfunction in Liver Transplant Recipients and Analysis of Risk Factors. *Liver Transplantation* **2010,** *16* (8), 943-949.

2. van Rijn, R.; Schurink, I. J.; de Vries, Y.; van den Berg, A. P.; Cerisuelo, M. C.; Murad, S. D.; Erdmann, J. I.; Gilbo, N.; de Haas, R. J.; Heaton, N.; et al., Hypothermic machine perfusion in liver transplantation — A randomized trial. *New England journal of medicine* **2021,** *384* (15), 1391‐1401.

3. Dwyer, M. E.; Dwyer, J. T.; Cannon, G. M., Jr.; Stephany, H. A.; Schneck, F. X.; Ost, M. C., The Clavien-Dindo Classification of Surgical Complications is Not a Statistically Reliable System for Grading Morbidity in Pediatric Urology. *J Urol* **2016,** *195* (2), 460-4.

4. Weeder, P. D.; van Rijn, R.; Porte, R. J., Machine perfusion in liver transplantation as a tool to prevent non-anastomotic biliary strictures: Rationale, current evidence and future directions. *J Hepatol* **2015,** *63* (1), 265-75.

5. de Vries, Y.; von Meijenfeldt, F. A.; Porte, R. J., Post-transplant cholangiopathy: Classification, pathogenesis, and preventive strategies. *Biochimica et biophysica acta. Molecular basis of disease* **2018,** *1864* (4 Pt B), 1507-1515.

6. Mergental, H.; Laing, R. W.; Kirkham, A. J.; Perera, M.; Boteon, Y. L.; Attard, J.; Barton, D.; Curbishley, S.; Wilkhu, M.; Neil, D. A. H.; Hübscher, S. G.; Muiesan, P.; Isaac, J. R.; Roberts, K. J.; Abradelo, M.; Schlegel, A.; Ferguson, J.; Cilliers, H.; Bion, J.; Adams, D. H.; Morris, C.; Friend, P. J.; Yap, C.; Afford, S. C.; Mirza, D. F., Transplantation of discarded livers following viability testing with normothermic machine perfusion. *Nature communications* **2020,** *11* (1), 2939.

7. Gaurav, R.; Butler, A. J.; Kosmoliaptsis, V.; Mumford, L.; Fear, C.; Swift, L.; Fedotovs, A.; Upponi, S.; Khwaja, S.; Richards, J.; Allison, M.; Watson, C. J. E., Liver Transplantation Outcomes From Controlled Circulatory Death Donors: SCS vs in situ NRP vs ex situ NMP. *Annals of surgery* **2022,** *275* (6), 1156-1164.

8. van Rijn, R.; Karimian, N.; Matton, A. P. M.; Burlage, L. C.; Westerkamp, A. C.; van den Berg, A. P.; de Kleine, R. H. J.; de Boer, M. T.; Lisman, T.; Porte, R. J., Dual hypothermic oxygenated machine perfusion in liver transplants donated after circulatory death. *The British journal of surgery* **2017,** *104* (7), 907-917.

9. Patrono, D.; Surra, A.; Catalano, G.; Rizza, G.; Berchialla, P.; Martini, S.; Tandoi, F.; Lupo, F.; Mirabella, S.; Stratta, C.; Salizzoni, M.; Romagnoli, R., Hypothermic Oxygenated Machine Perfusion of Liver Grafts from Brain-Dead Donors. *Sci Rep* **2019,** *9* (1), 9337.

# Figure legends


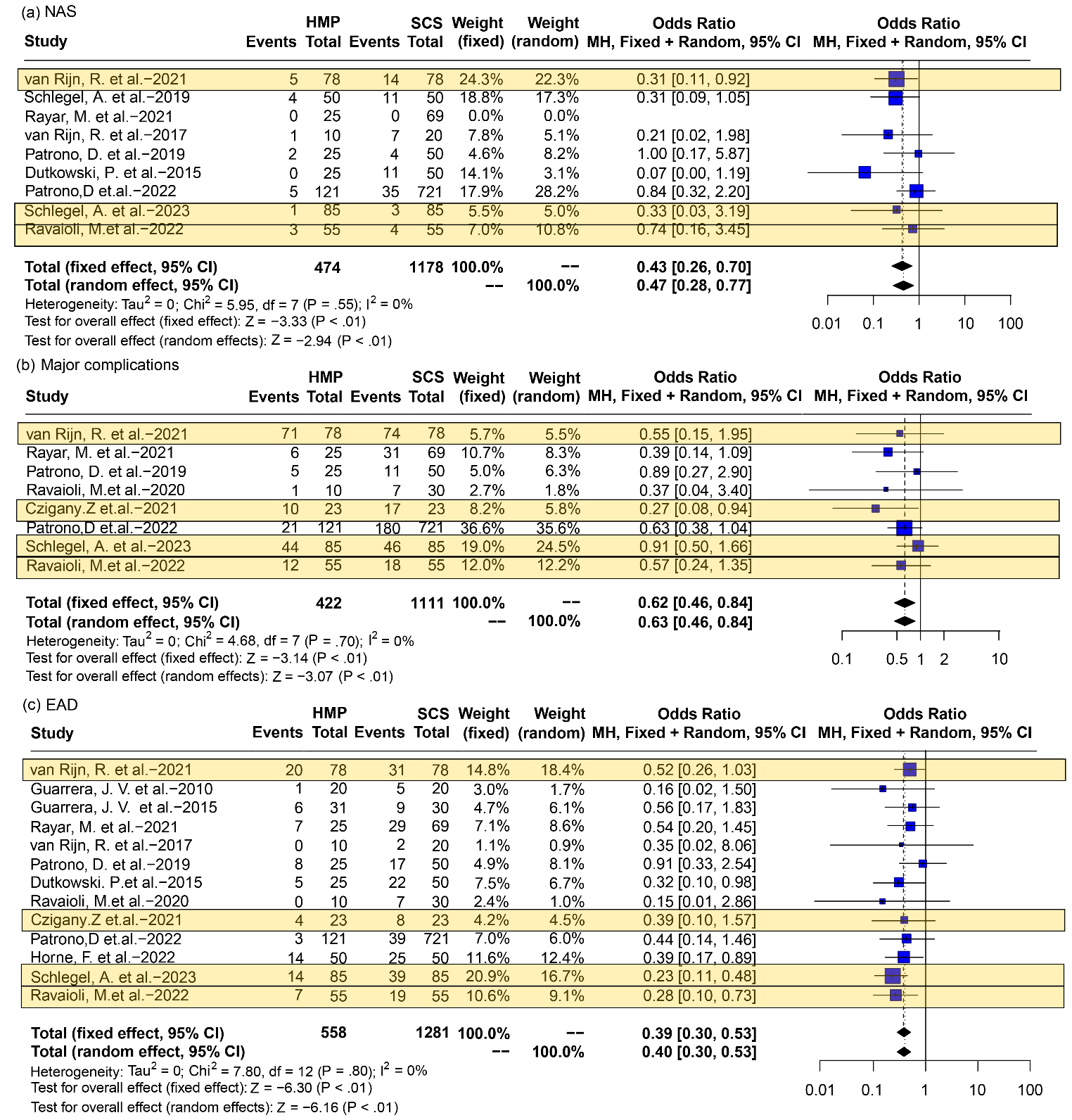


**Figure S1. Forest plots on NAS(a), major complications(b), EAD(c) in OLT(all donor types) after HMP compared with SCS.** CI, confidence interval; ECD, extended/expanded criteria donor; EAD, early allograft dysfunction; NAS, non-anastomotic biliary stricture; SCS, static cold storage; HMP, hypothermic machine perfusion. RCT data are highlighted in yellow.


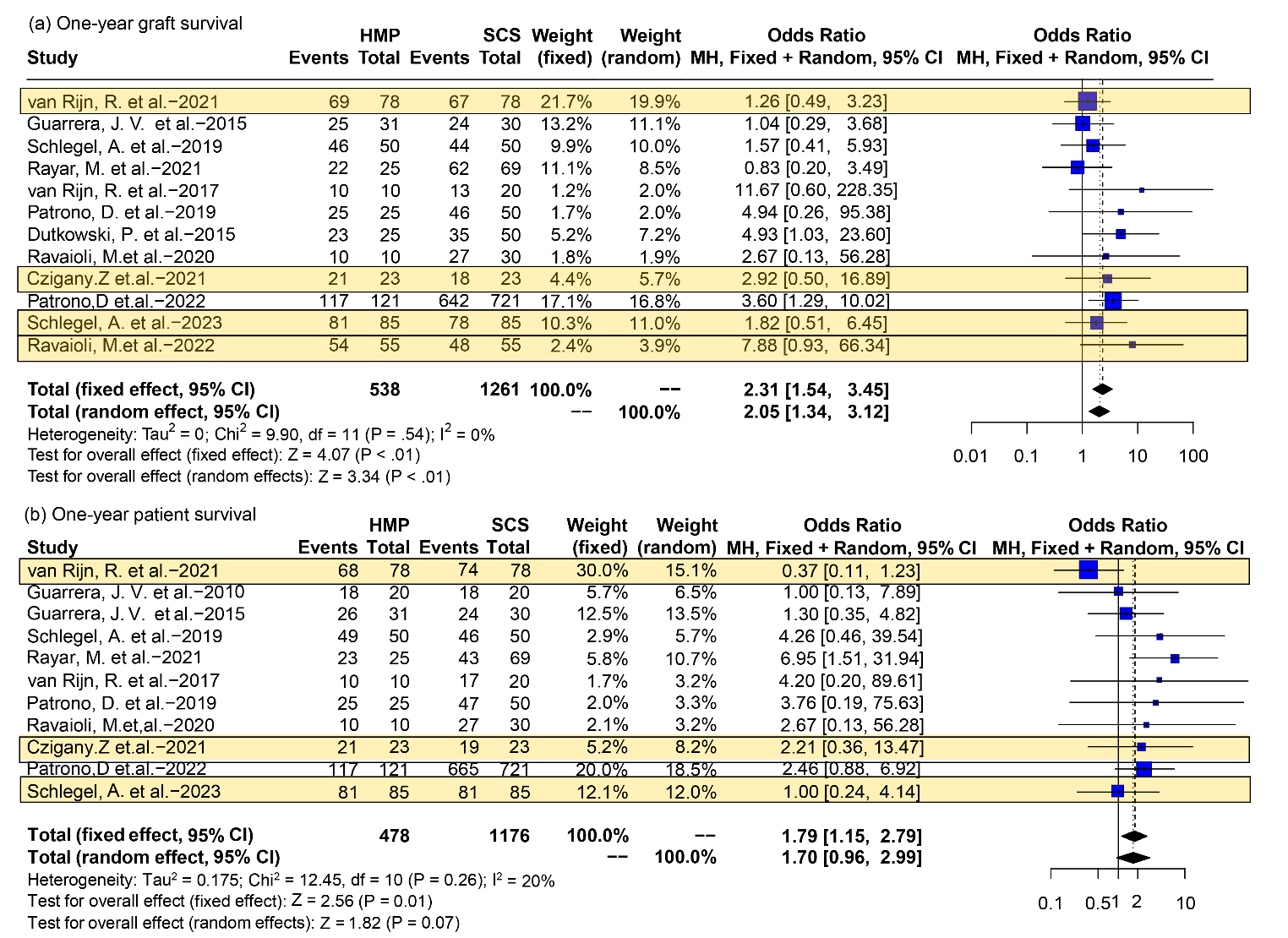


**Figure S2. Forest plots on one-year graft survival(a), one-year patient survival(b) in OLT(all donor types) after HMP compared with SCS.** CI, confidence interval; SCS, static cold storage; HMP, hypothermic machine perfusion. RCT data are highlighted in yellow.


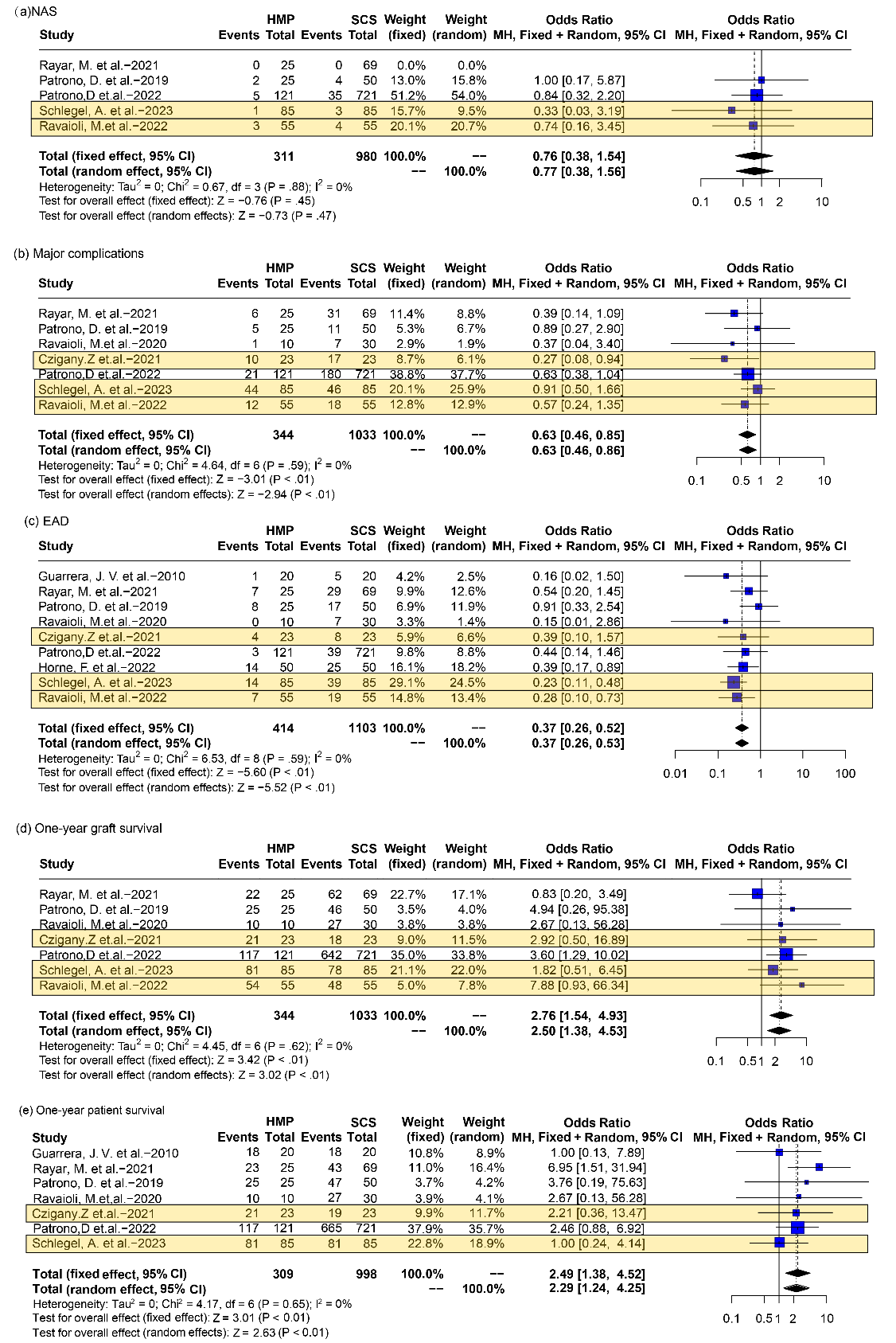


**Figure S3. Forest plots on NAS(a), major complications(b), EAD(c), one-year graft survival(d), one-year patient survival(e) in DBD-OLT after HMP compared with SCS.** CI, confidence interval; DBD, donor after brainstem death; EAD, early allograft dysfunction; NAS, non-anastomotic biliary stricture; SCS, static cold storage; HMP, hypothermic machine perfusion. RCT data are highlighted in yellow.

**
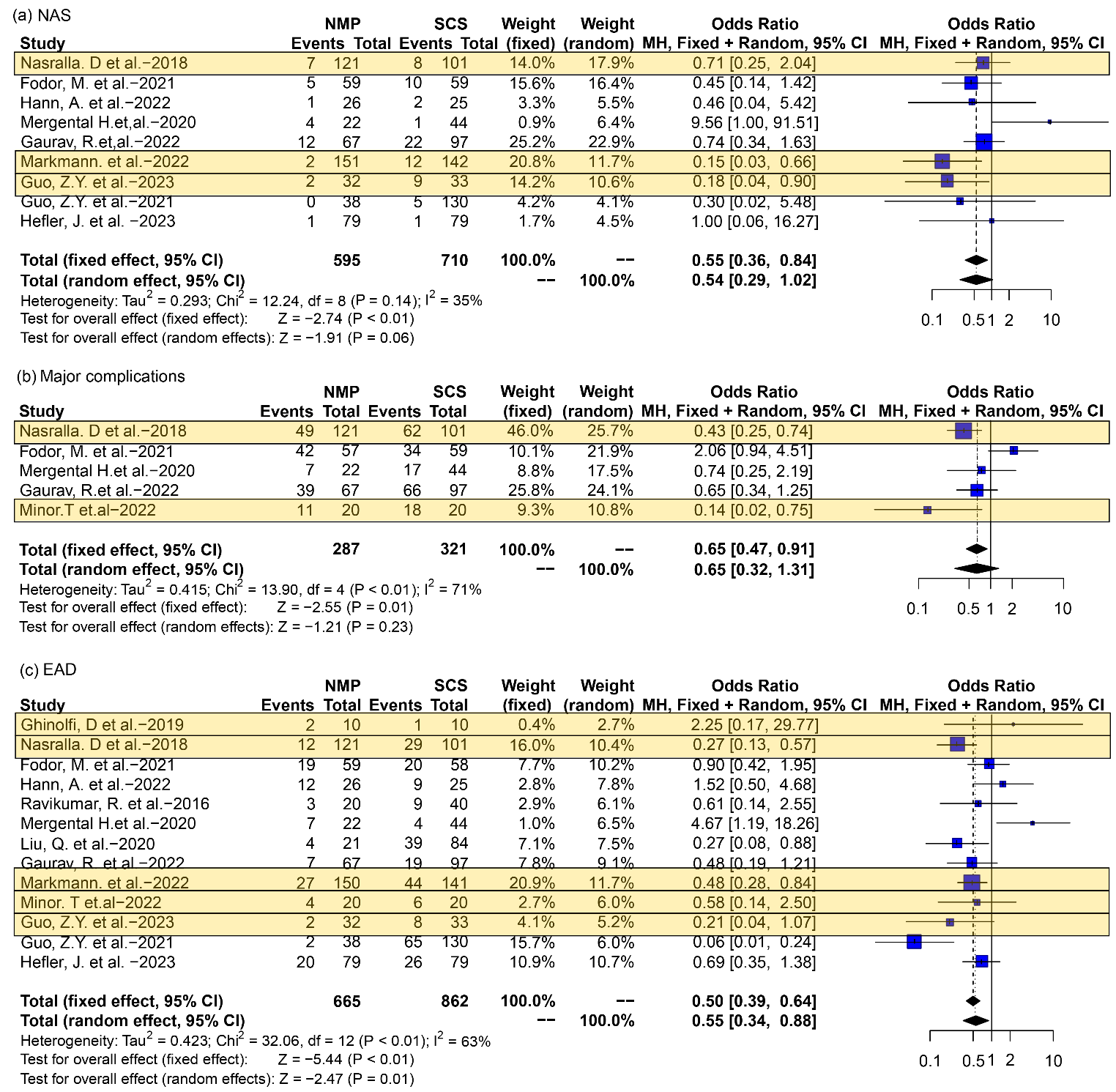
Figure S4. Forest plots on NAS(a), major complications(b), EAD(c) in OLT(all donor types) after NMP compared with SCS.** CI, confidence interval; ECD, extended/expanded criteria donor; EAD, early allograft dysfunction; NAS, non-anastomotic biliary stricture; SCS, static cold storage; NMP, normothermic machine perfusion. RCT data are highlighted in yellow. Data of Hefler’study were extracted from matching 1:1 cohorts

**
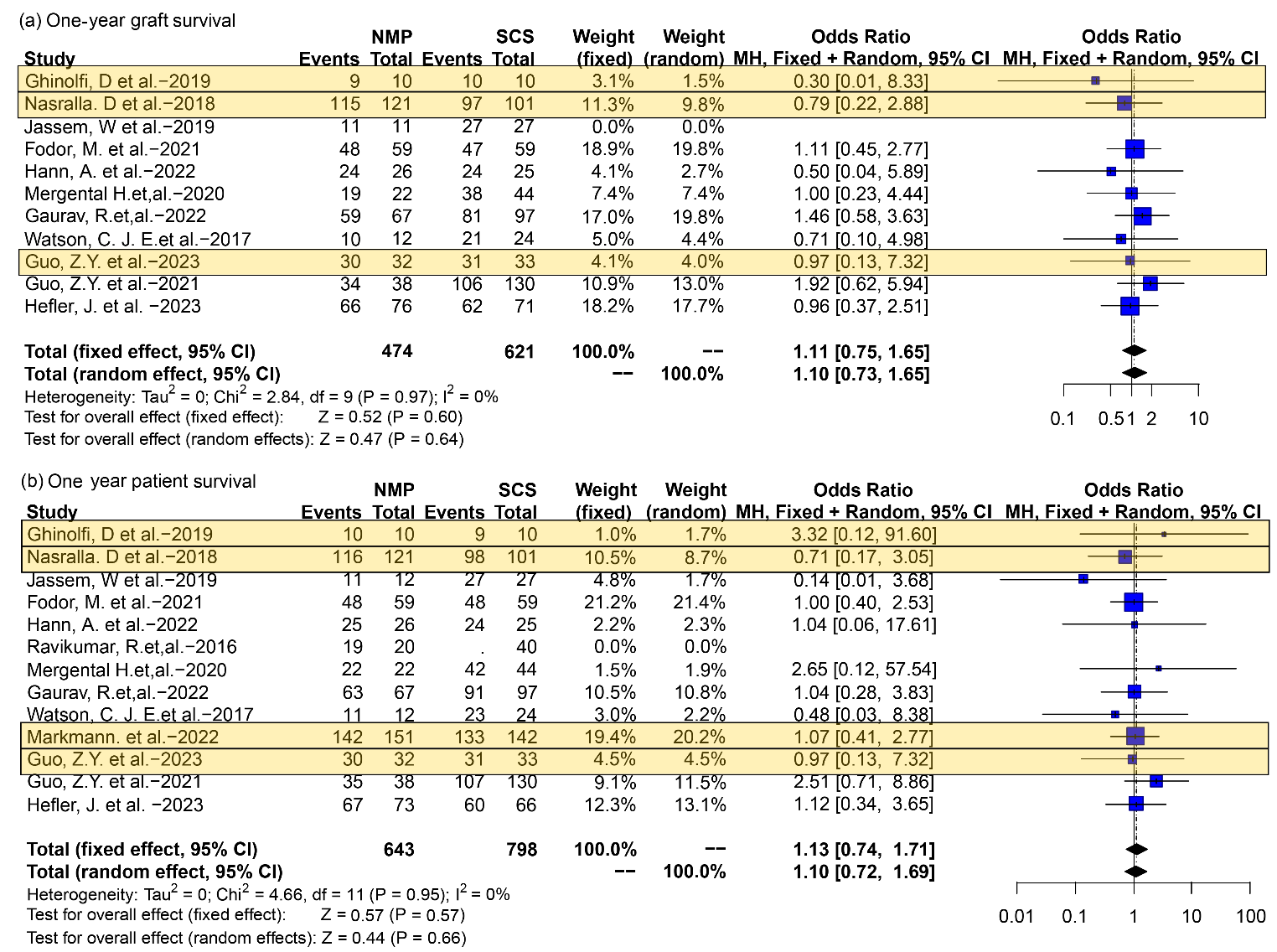
**

**Figure S5. Forest plots on one-year graft survival(a), one-year patient survival(b) in OLT(all donor types) after NMP compared with SCS.** CI, confidence interval; SCS, static cold storage; NMP, normothermic machine perfusion. RCT data are highlighted in yellow. Data of Hefler’study were extracted from matching 1:1 cohorts


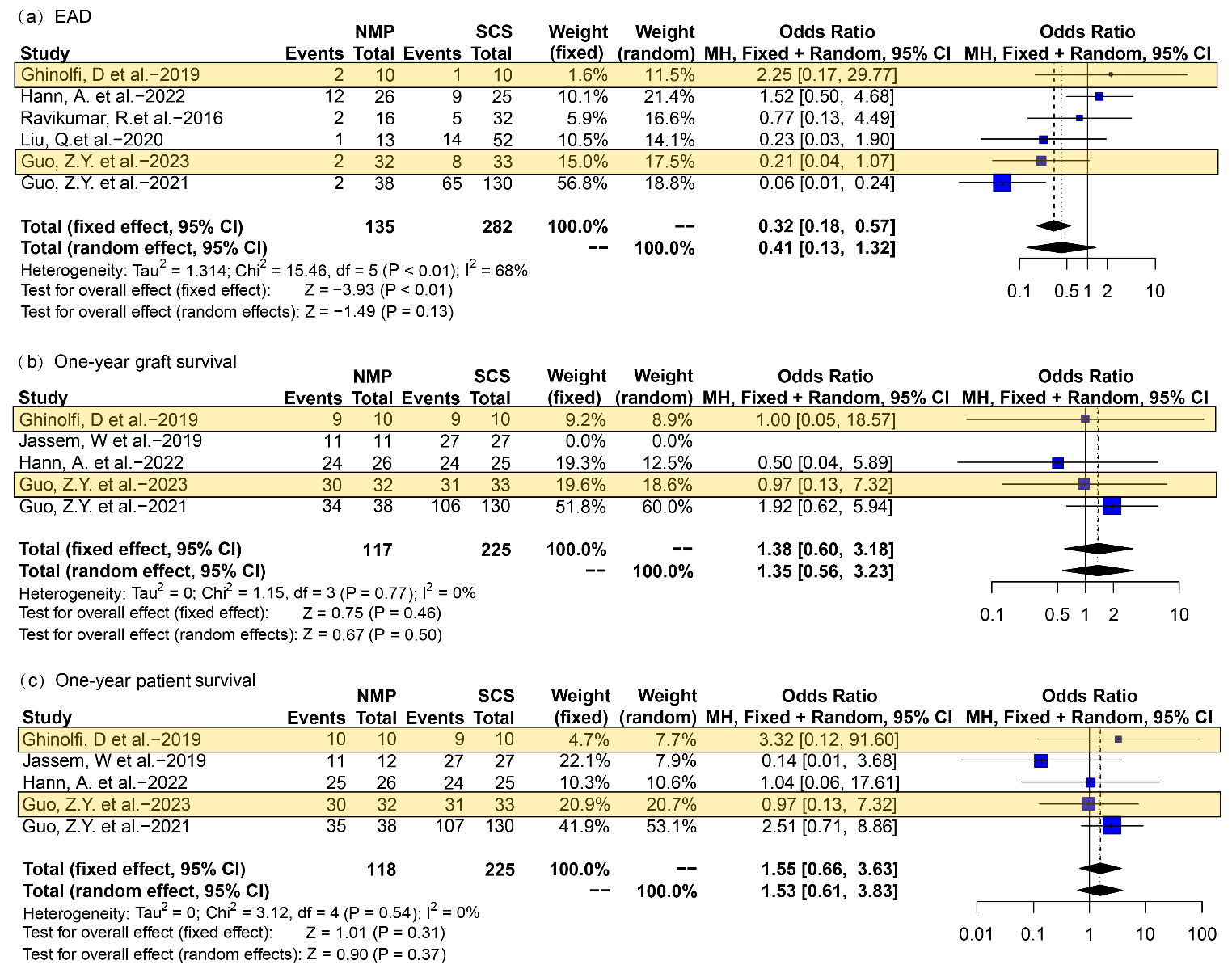


**Figure S6. Forest plots on EAD(a), one-year graft survival(b), one-year patient survival(c) in DBD-OLT after NMP compared with SCS.** CI, confidence interval; DBD, donor after brainstem death ;EAD, early allograft dysfunction; SCS, static cold storage; NMP, normothermic machine perfusion. RCT data are highlighted in yellow.

**
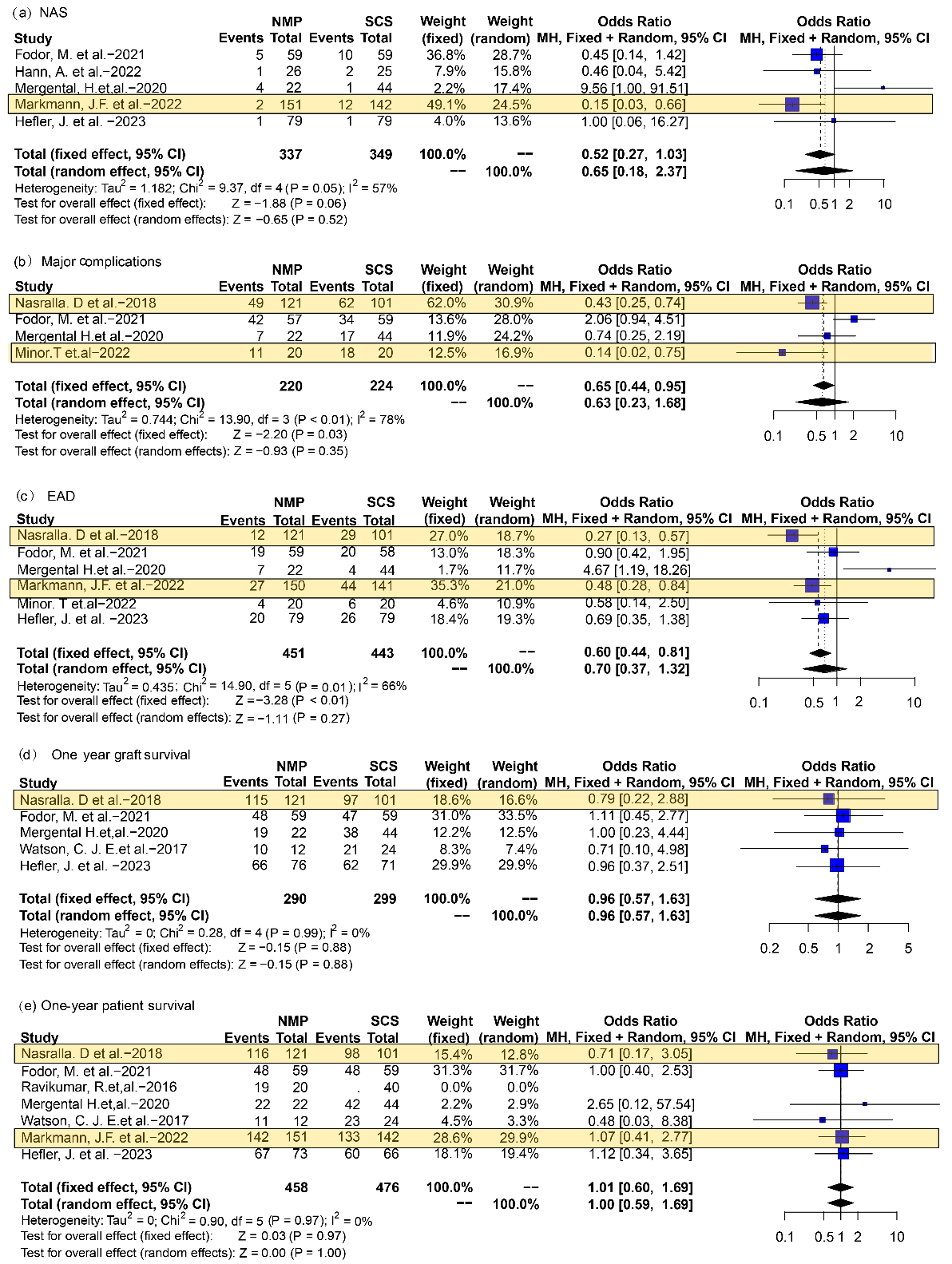
**

**Figure S7. Forest plots on NAS(a), major complications(b), EAD(c), one-year graft survival(d), one-year patient survival(e) in studies including both DBD-OLT &DCD-OLT after NMP compared with SCS.** CI, confidence interval; DBD, donor after brainstem death; DCD, donation after circulatory death; EAD, early allograft dysfunction; NAS, non-anastomotic biliary stricture; SCS, static cold storage; NMP, normothermic machine perfusion. RCT data are highlighted in yellow. Data of Hefler’study were extracted from matching 1:1 cohorts

**
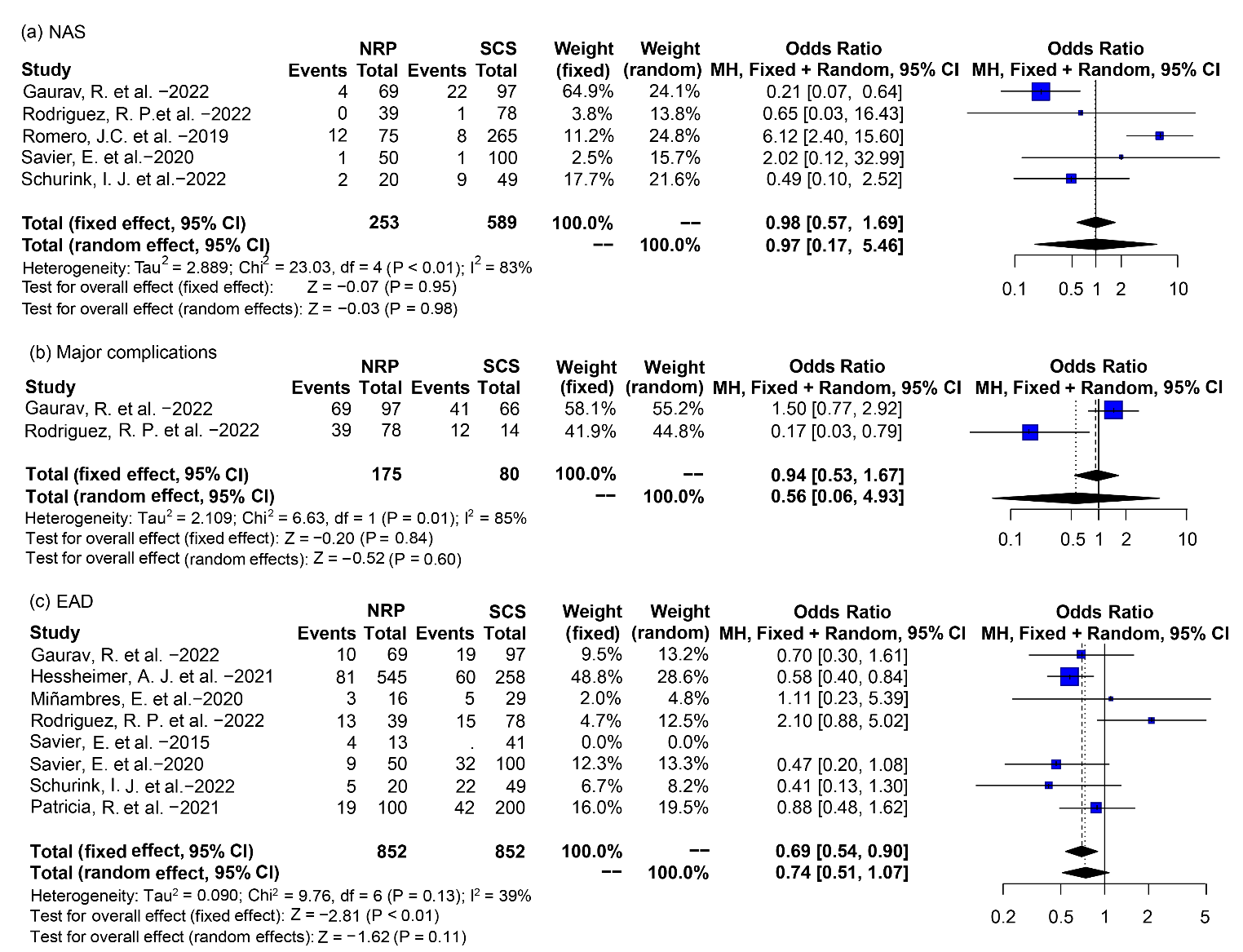
Figure S8. Forest plots on NAS(a), major complications(b), EAD(c) in both cDCD-OLT and uDCD-OLT after NRP compared with SCS.** CI, confidence interval; ECD, extended/expanded criteria donor; EAD, early allograft dysfunction; NAS, non-anastomotic biliary stricture; SCS, static cold storage; NRP, normothermic regional perfusion.

**
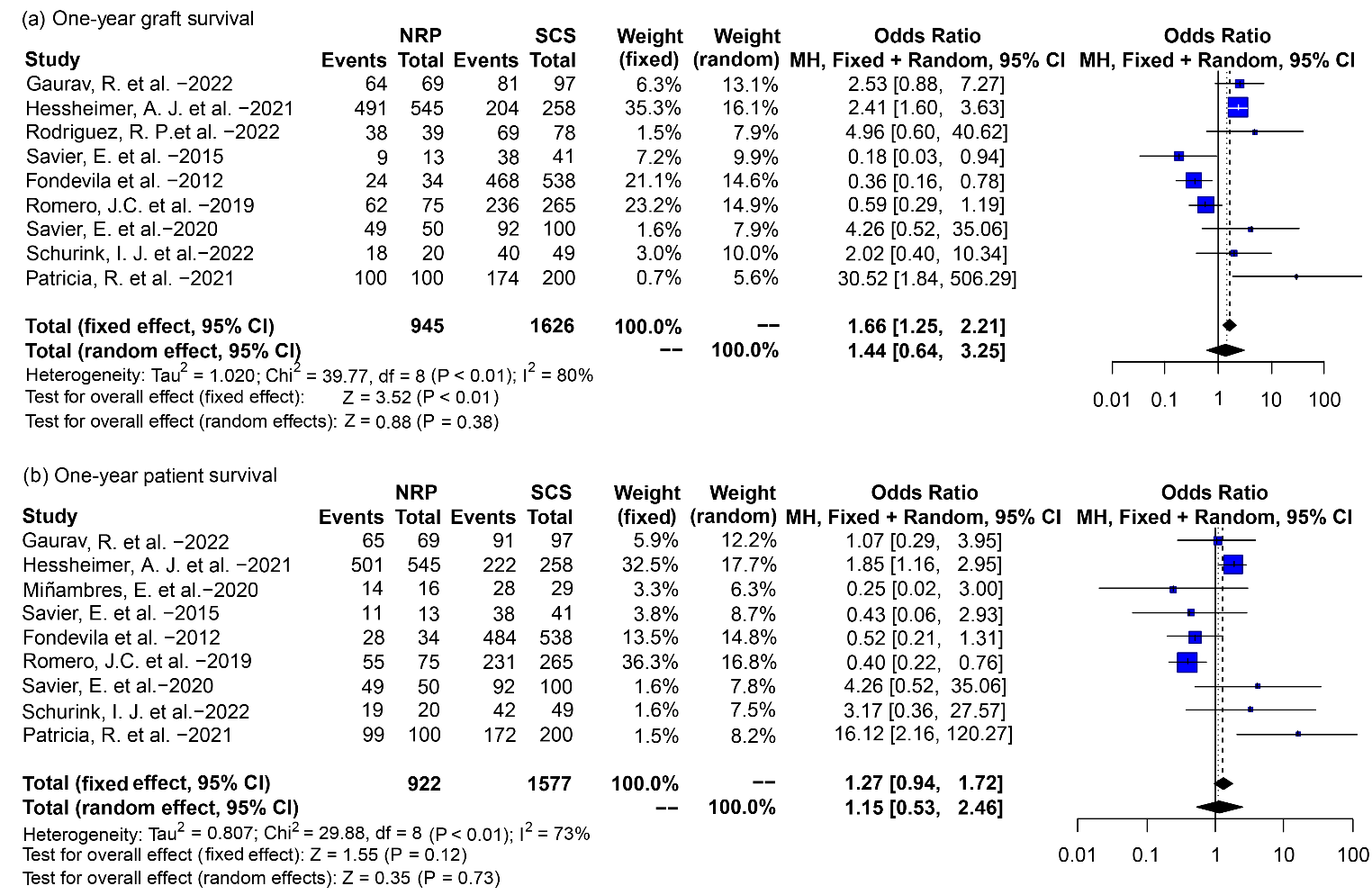
Figure S9. Forest plots on one-year graft survival(a), one-year patient survival(b) in both cDCD-OLT and uDCD-OLT after NRP compared with SCS.** CI, confidence interval; SCS, static cold storage; NRP, normothermic regional perfusion

**
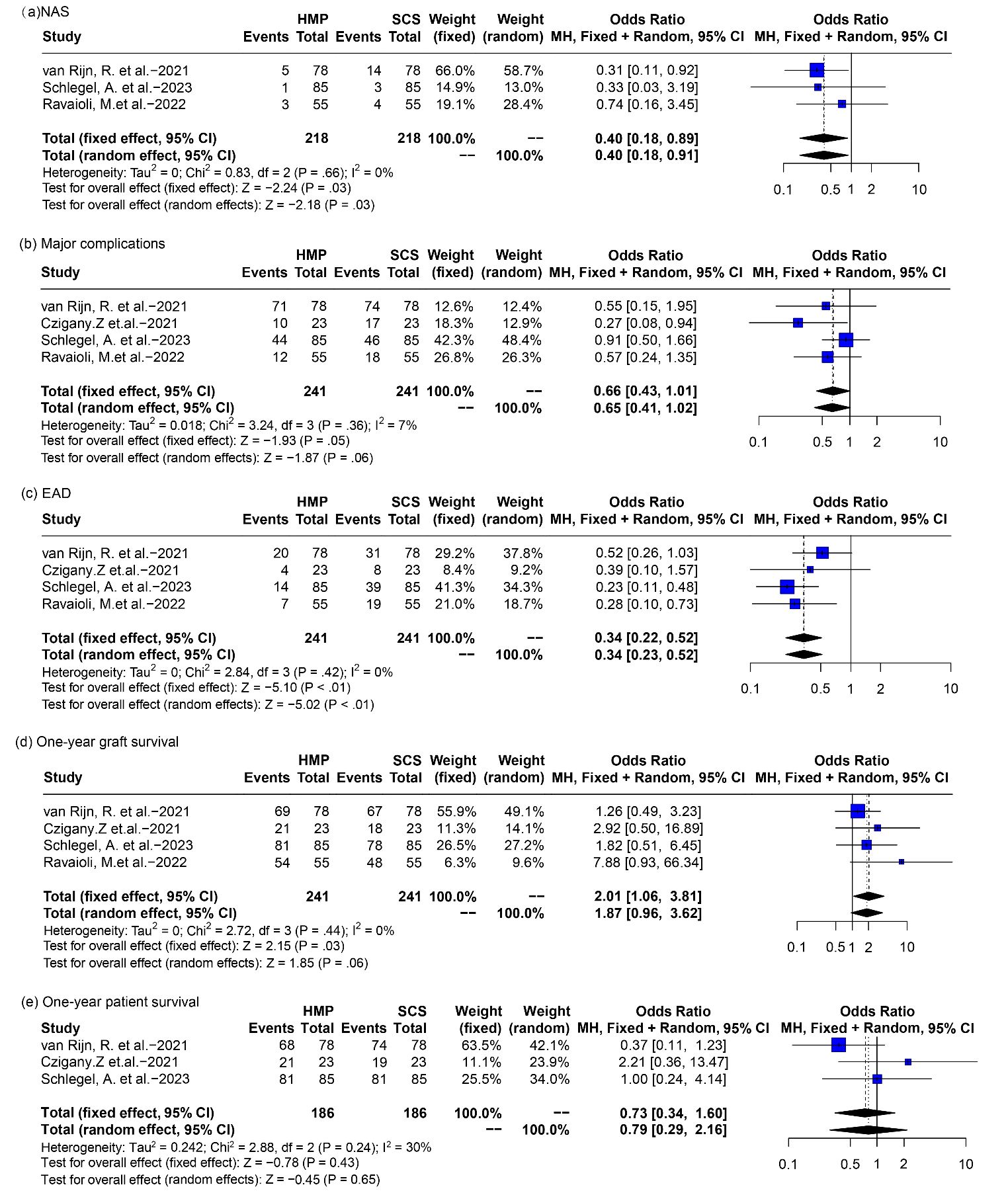
Figure S10. Forest plots on NAS(a), major complications(b), EAD(c), one-year graft survival(d), one-year patient survival(e) in OLT after HMP compared with SCS in RCTs.** CI, confidence interval; EAD, early allograft dysfunction; NAS, non-anastomotic biliary stricture; SCS, static cold storage; HMP, hypothermic machine perfusion

**
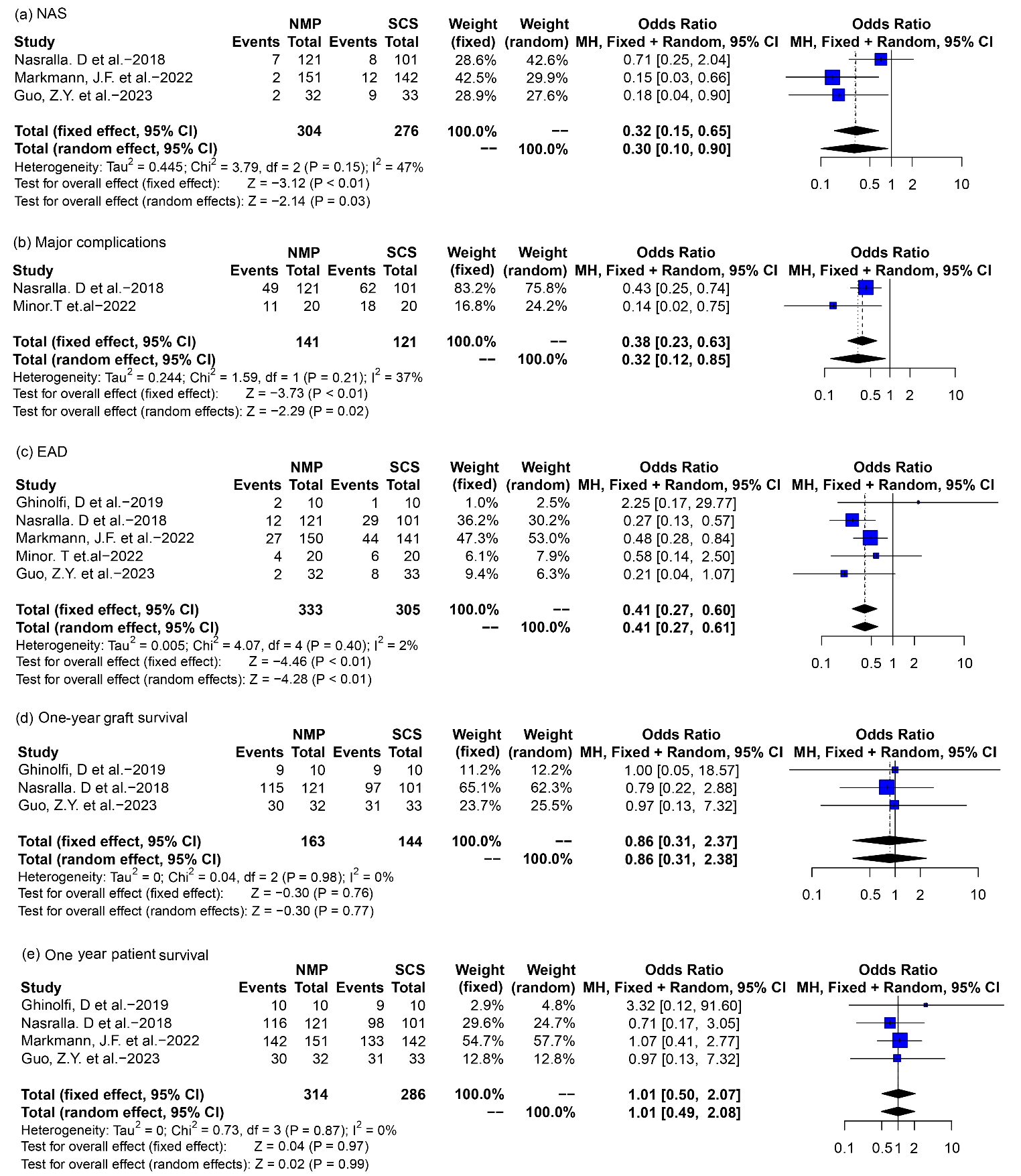
Figure S11. Forest plots on NAS(a), major complications(b), EAD(c), one-year graft survival(d), one-year patient survival(e) in OLT after NMP compared with SCS in RCTs.** CI, confidence interval; EAD, early allograft dysfunction; NAS, non-anastomotic biliary stricture; SCS, static cold storage; NMP, normothermic machine perfusion

**
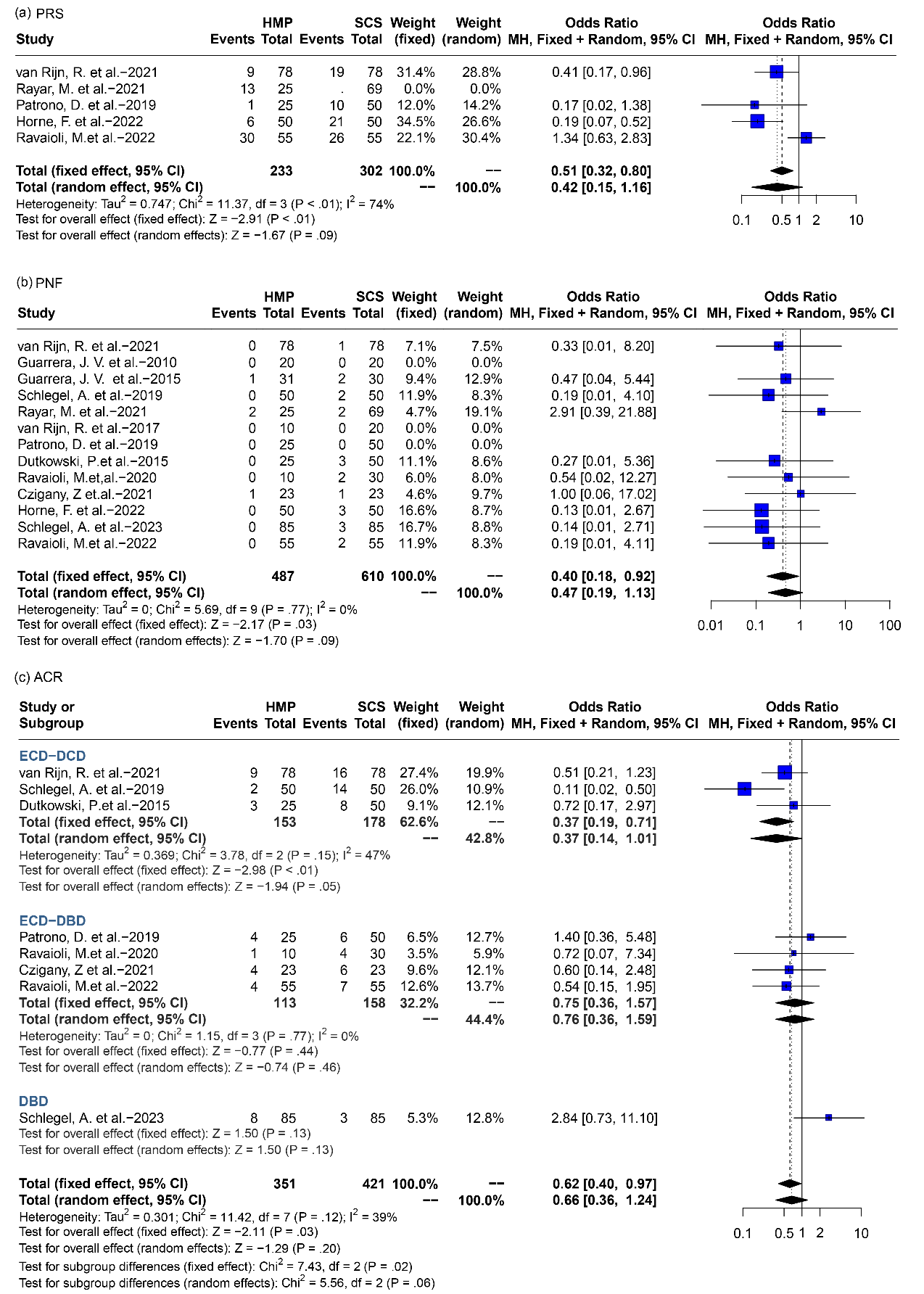
**

**Figure S12. Forest plots on PRS(a), PNF(b), ACR(c) in OLT after HMP compared with SCS.** CI, confidence interval; PRS, post-reperfusion syndrome; PNF, primary non-function; ACR, acute cellular rejection; SCS, static cold storage; HMP, hypothermic machine perfusion

**
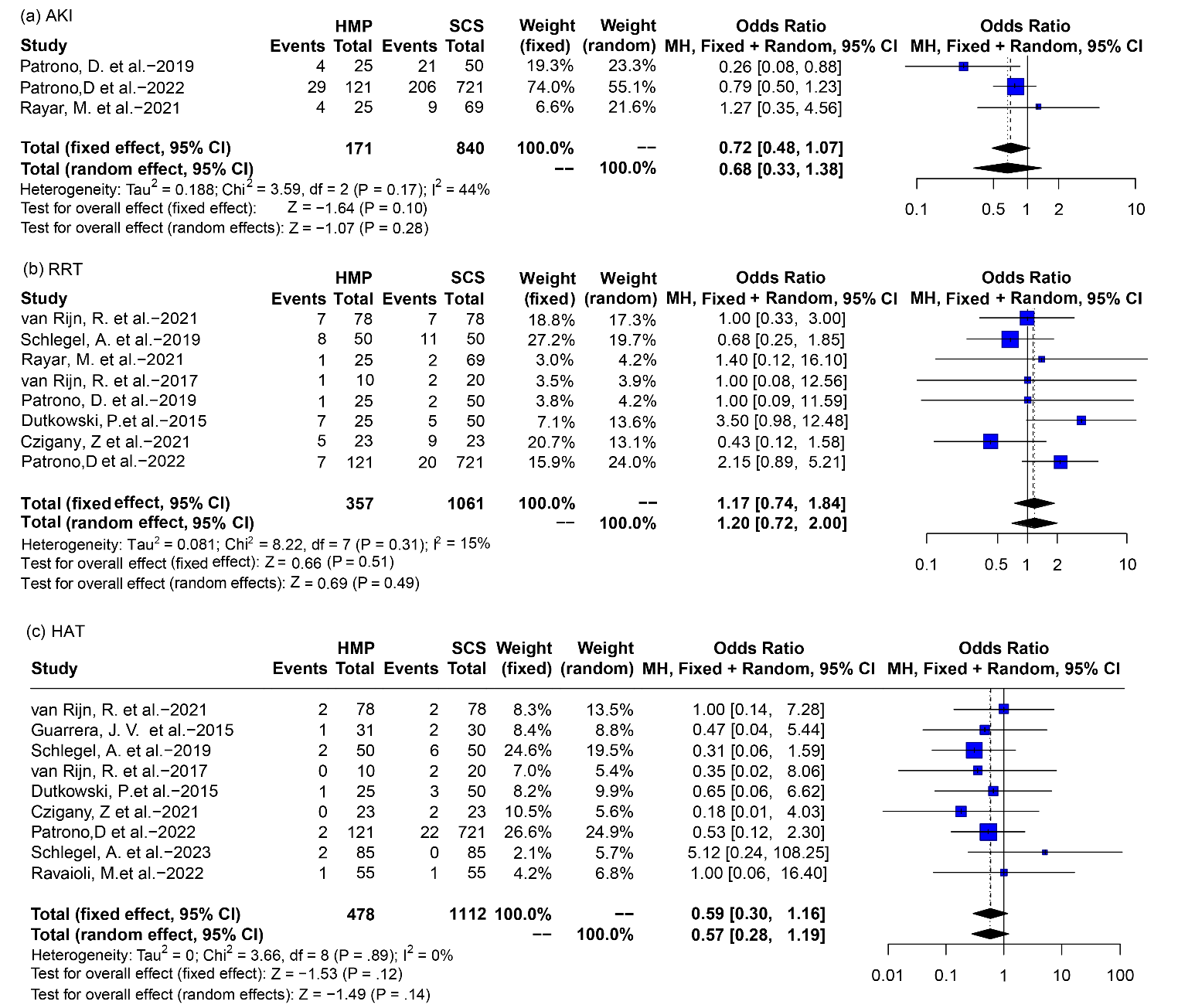
Figure S13. Forest plots on AKI(a), RRT(b), HAT(c) in OLT after HMP compared with SCS.** CI, confidence interval; AKI, acute kidney injury; RRT, renal replacement therapy; HAT, hepatic artery thrombosis; SCS, static cold storage; HMP, hypothermic machine perfusion

**
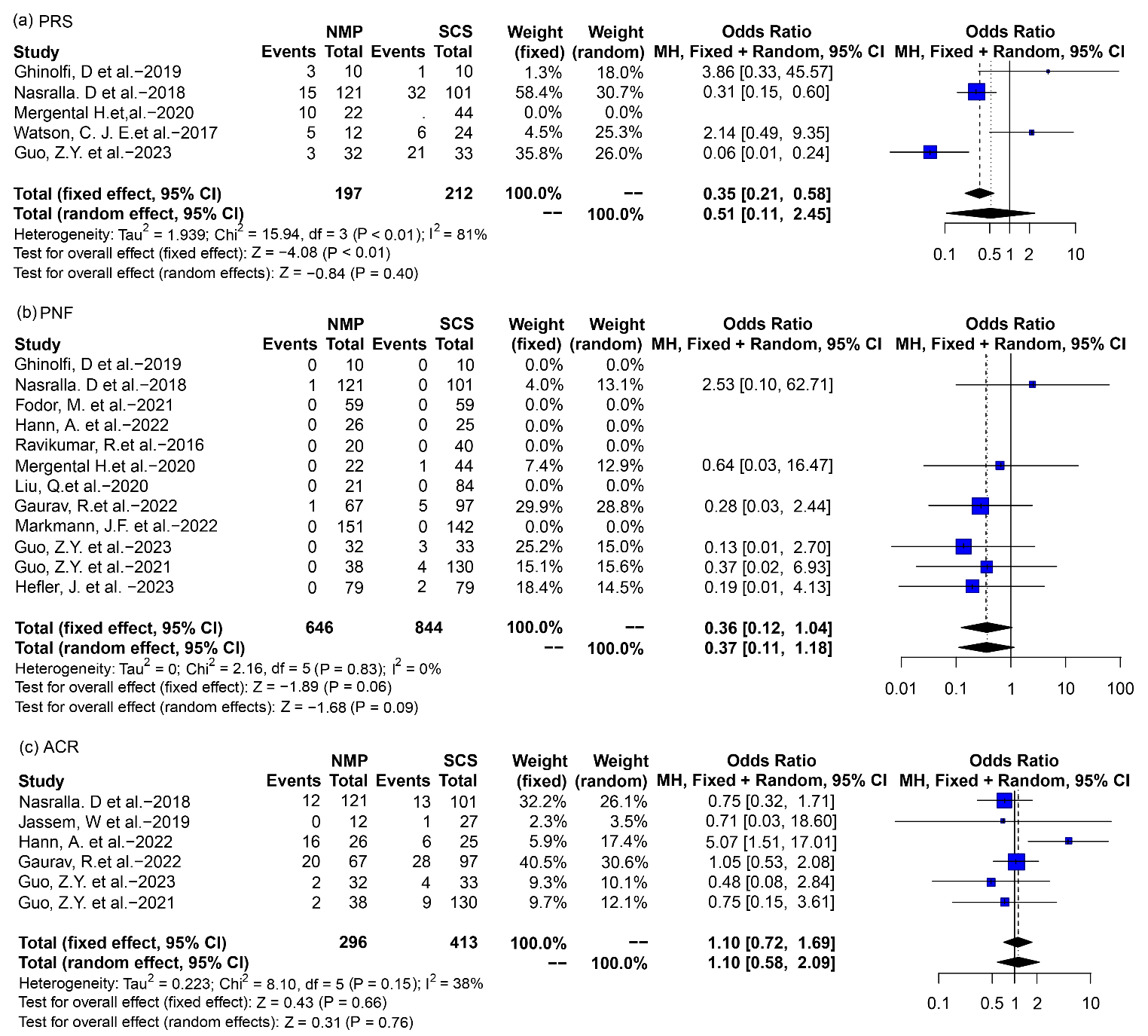
Figure S14. Forest plots on PRS(a), PNF(b), ACR(c) in OLT after NMP compared with SCS.** CI, confidence interval; PRS, post-reperfusion syndrome; PNF, primary non-function; ACR, acute cellular rejection; SCS, static cold storage; NMP, normothermic machine perfusion

**
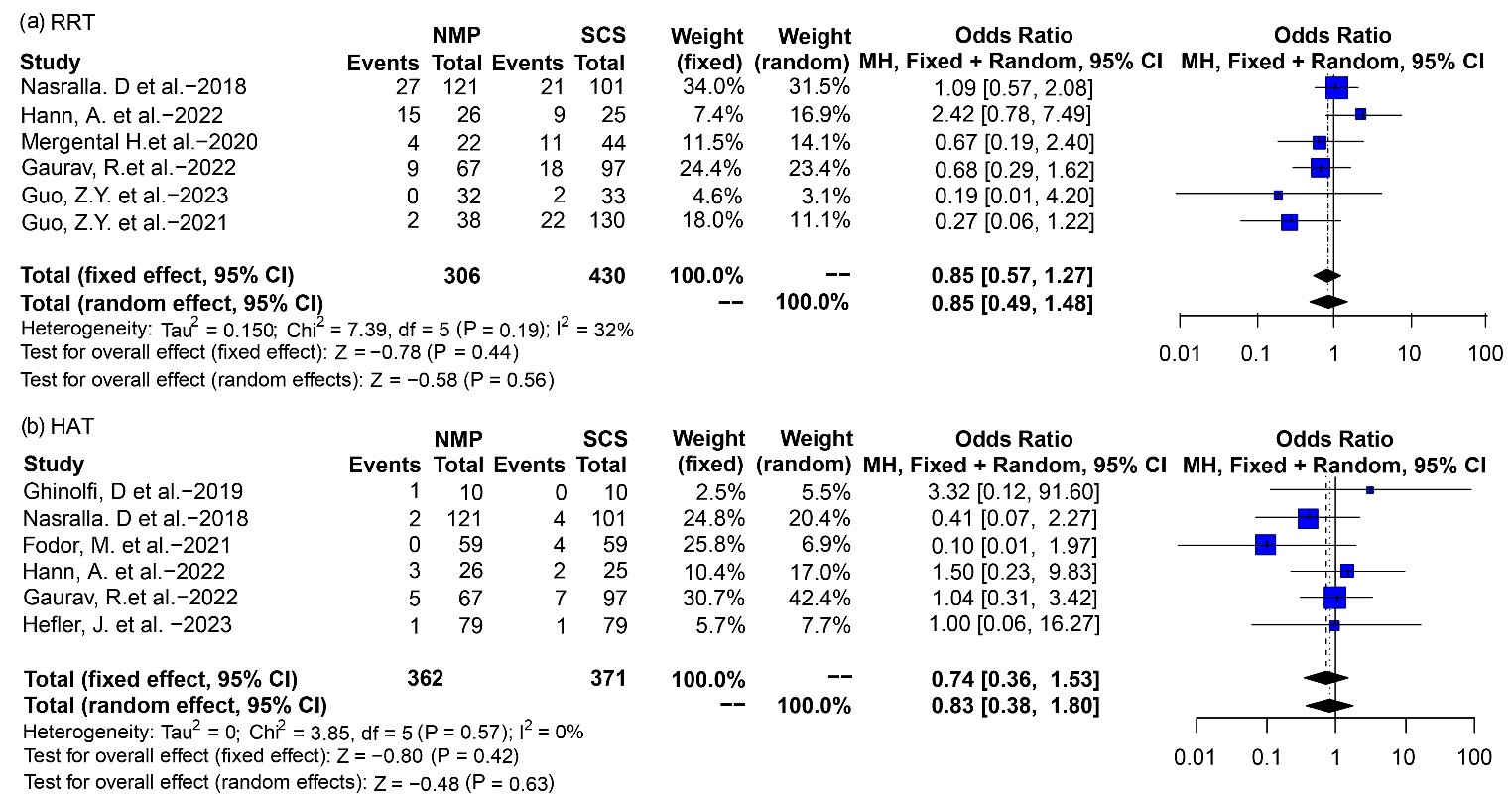
Figure S15. Forest plots on RRT(a), HAT(b) in OLT after NMP compared with SCS.** CI, confidence interval; RRT, renal replacement therapy; HAT, hepatic artery thrombosis; SCS, static cold storage; NMP, normothermic machine perfusion

**
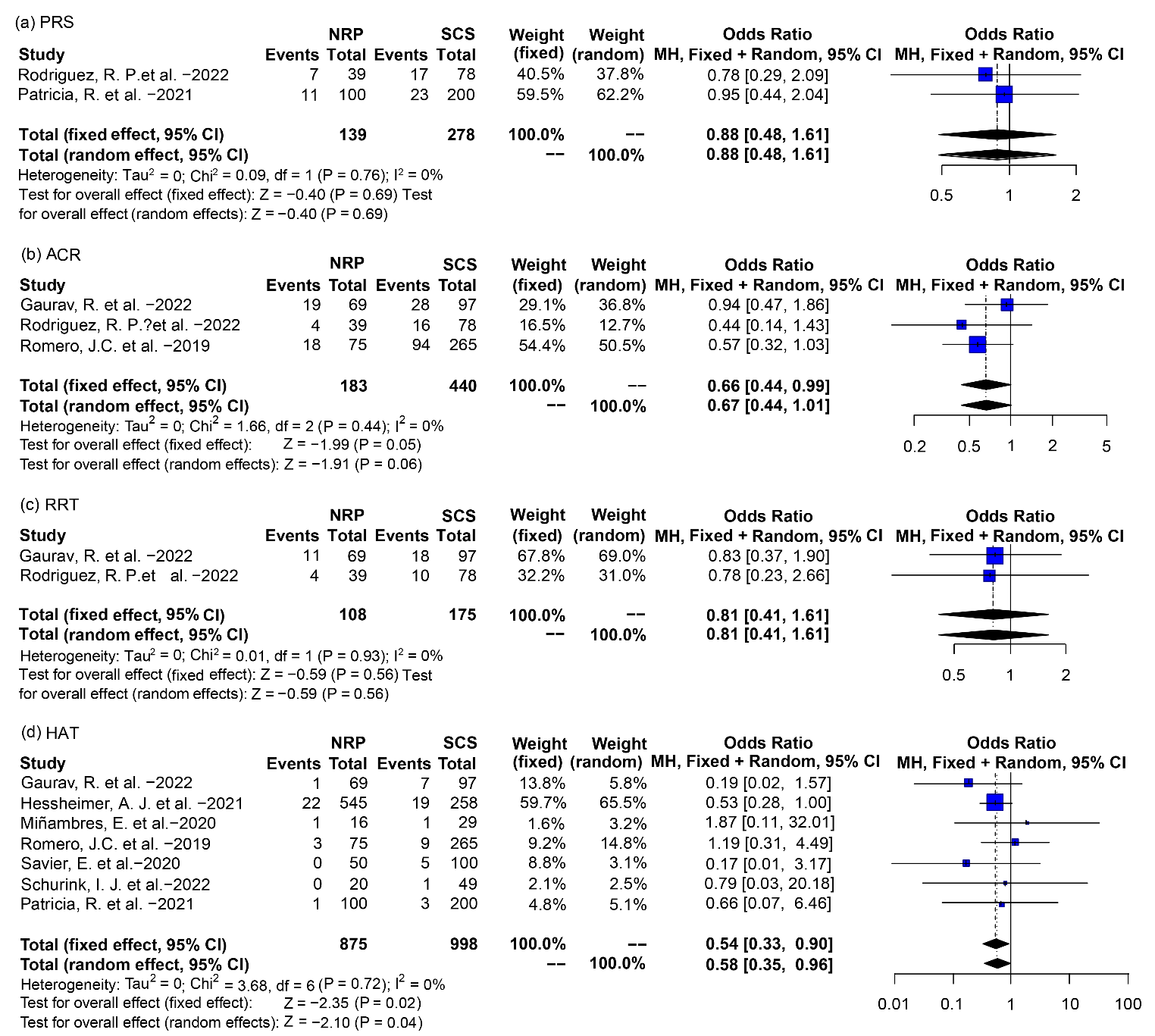
Figure S16. Forest plots on PRS(a), ACR(b), RRT(c), HAT(d) in OLT after NRP compared with SCS.** CI, confidence interval; PRS, post-reperfusion syndrome; ACR, acute cellular rejection; RRT, renal replacement therapy; HAT, hepatic artery thrombosis; SCS, static cold storage; NRP, normothermic regional perfusion
